# Bias Dependence of the Transition State of the Hydrogen Evolution Reaction

**DOI:** 10.1021/jacs.4c18638

**Published:** 2025-02-03

**Authors:** José M. Gisbert-González, Carlos G. Rodellar, Jody Druce, Eduardo Ortega, Beatriz Roldan Cuenya, Sebastian Z. Oener

**Affiliations:** Department of Interface Science, Fritz-Haber Institute of the Max Planck Society, Berlin 14195, Germany

## Abstract

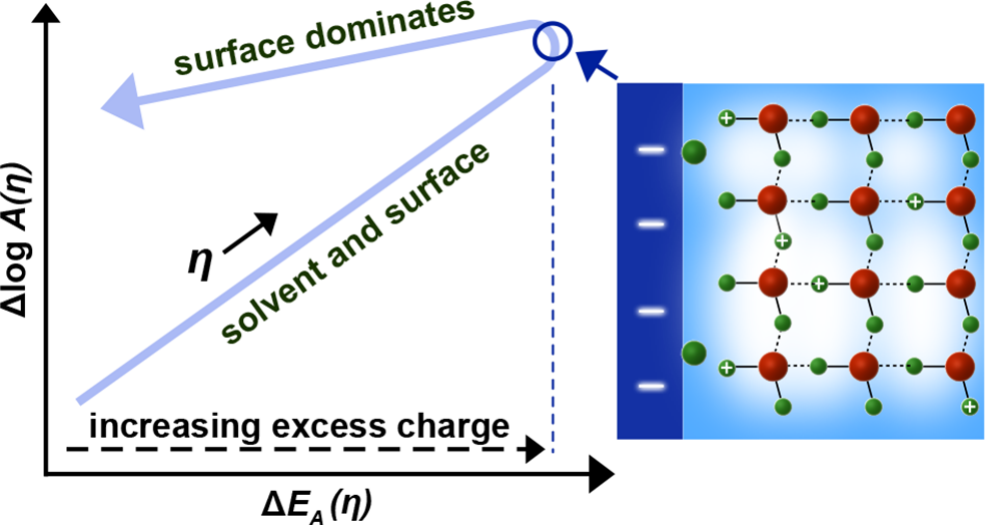

The hydrogen evolution reaction (HER) is one of the most prominent electrocatalytic reactions of green energy transition. However, the kinetics across materials and electrolyte pH and the impact of hydrogen coverage at high current densities remain poorly understood. Here, we study the HER kinetics over a large set of nanoparticle catalysts in industrially relevant acidic and alkaline membrane electrode assemblies that are only operated with pure water humidified gases. We discover distinct kinetic fingerprints between the iron triad (Fe, Ni, Co), coinage (Au, Cu, Ag), and platinum group metals (Ir, Pt, Pd, Rh). Importantly, the applied bias changes not only the activation energy (*E*_A_) but also the pre-exponential factor (*A*). We interpret these changes as entropic changes in the interfacial solvent that differ between acid and base and entropic changes on the surface due to a changing hydrogen coverage. Finally, we observe that anions can induce Butler–Volmer behavior for the coinage metals in acid. Our results provide a new foundation to understand HER kinetics and, more broadly, highlight the pressing need to update common understanding of basic concepts in the field of electrocatalysis.

## Introduction

The hydrogen evolution reaction (HER) is a key electrocatalytic reaction of the green energy transition. It uses green electricity and electrocatalysts to convert protons from aqueous electrolytes into green hydrogen. As such, it serves as the prototypical example of all inner-sphere proton-coupled electron transfer reactions in aqueous media. Despite this, the reasons behind the vastly different activities among different metal catalysts remain unclear. Furthermore, the sluggish kinetics in alkaline environments compared to acidic ones are not understood. Other questions pertain to the impact of increasing hydrogen coverage at higher current densities. These significant knowledge gaps limit rational designs and improvements of alkaline and acidic electrolyzers that are operated at industrially relevant current densities.

HER kinetics are commonly described using the hydrogen binding energy as the primary activity descriptor.^1^ According to the Sabatier principle, the ideal catalyst with the highest activity appears on top of a volcano activity plot, as it does not bind the hydrogen too weakly, nor too strongly. These models generally assume that the initial proton discharge step is barrier-less,^1^ that only one type of hydrogen participates in the reaction and that it does not change its coverage with bias. New models can capture bias dependent configurations and free energy changes of surface intermediates^2^ and short-range electrostatic interactions in the solvent^3,4^ and, thus, are regularly used to estimate reorganization parameters that impact the activation enthalpy in the exponent of the rate law. However, current computational power is insufficient to capture long-range electric field effects across the extended hydrogen bond network and the impact on the activation entropy and transition states in catalysis more broadly.^5−9^ Conversely, across the literature, inner-sphere reactivity is essentially modeled with outer-sphere type theory, where the bias reduces the activation energy (*E*_A_) and the activation entropy (Δ*S*) in the pre-exponential factor (*A*) of the Eyring–Evans–Polanyi equation^10,11^ stays constant.

These computationally constrained models are increasingly challenged.^12,13^ Catalyst surfaces on the descending volcano branch (such as for W, Mo, Ti) are covered with amorphous oxides under operation,^14^ challenging reliable density functional theory (DFT).^12^ Further, depending on the bias and binding site, different types of (under- or overpotential deposited) hydrogen exist that can repel each other and compete for adsorption or coparticipate in the reaction.^15^ Finally, barriers of ion (de)solvation have been neglected in early work.^1^ Pt group metals (PGMs) possess long-range overlap between the metal 5d-band and the 1s orbital of the solvated H^+^ in acid. As a result, the bias can directly reduce the activation enthalpy,^6^ mirroring outer-sphere kinetics.^16,17^ However, many 3d-band metals, such as Ni and Co, possess only weak overlap in acid, let alone the coinage metals, which are devoid of any substantial hydrogen binding ability.^12^ The situation is even less clear for the interfacial solvation of hydroxide ions, which is thought to be critical to understand the suppressed HER activity in alkaline electrolyte (2H_2_O + 2e^–^ → H_2_ + 2OH^–^). Recently, we discovered shared entropy–enthalpy compensation effects across different electrocatalytic reactions in alkaline electrolyte and even for isolated water dissociation and ion solvation in bipolar membranes.^18,19^ We reasoned that an increasingly ordered hydrogen bond network leads to an effective delocalization of the ion in the transition state and, thus, an increasing activation entropy, albeit at the expense of raising the activation enthalpy. However, so far, there exists no comprehensive picture for the entropic changes that extends across the material phase space and, especially, to different electrolyte conditions. Importantly, gaining insights into entropic effects in the solvent not only for the fast PGMs, but, especially, the slow kinetics on the coinage metals is important to understand the electrocatalytic CO_2_ reduction reaction on the same catalyst surfaces. For example, Schreier and co-workers^20^ observed near-zero activation energies at applied bias and CO_2_ reduction rates that were strongly governed by a bias dependent activation entropy for imidazolium cation-modified Ag surfaces.

The rise of operando spectroscopy and microscopy has led to an unprecedented level of insight about material-specific factors that govern catalyst operation, such as (dynamic) crystal facets, step edges, undercoordinated sites and the chemical environment around the active sites. However, despite decades of research, the impact of the interfacial solvent and hydrogen coverage on HER kinetics remain unclear. Conversely, there is a clear need for comparative studies across the material phase space to draw general conclusions on the reaction mechanism, even if it means sacrificing material-specific resolution.

Here, we perform a comparative study on the temperature-dependent HER kinetics over a large set of metallic nanoparticle catalysts in acidic and alkaline membrane electrolyzers that are only operated with pure water humidified gases. When we extract the bias dependent activation entropy and enthalpy, we discover common kinetic fingerprints within each metal group of the iron triad (Ni, Fe, Co), coinage (Au, Cu, Ag) and platinum group metals (PGMs; Ir, Pt, Pd, Rh). Most importantly, except for the PGMs, none of the other groups display simple Butler–Volmer-type kinetics. Instead, the bias not only changes the activation energy, but also the activation entropy in the pre-exponential factor. Supported by the bias dependent (pseudo)-capacitance, we separate the activation entropy changes into changes in the interfacial solvent that differ between H^+^ and OH^–^ solvation, and changes on the catalyst surface that indicate how a bias dependent H coverage impacts HER kinetics. Finally, we observe that exposure of the pure-water systems to anions can induce Butler–Volmer type behavior for the coinage metals. These results are critical to understand the high H_2_ product selectivity in acid during the CO_2_ reduction on the same catalyst surfaces.

### Analyzing Steady-State HER Rates under High Mass Transport

HER catalyst kinetics are often analyzed by identifying and interpreting (seemingly) linear Tafel slopes (d log(*J*)dη^–1^) at a constant temperature. Related, it is often assumed that the exchange current density, *j*_0_, either obtained by interpolating from higher bias to equilibrium or by linear regression in the micropolarization region, informs on the “intrinsic” activity which is set solely by conditions at equilibrium. In these pictures, the bias simply reduces (increases) the activation energy of the forward (backward) reaction. Inherently, this approach is poorly suited to describe (bias dependent) changes in the inner-sphere of heterogeneous interfaces.

Temperature dependent Tafel slopes can inform on the enthalpic and entropic components of the charge transfer coefficient, as demonstrated by Conway for the HER in acid on sp-metals.^21−24^ However, this approach has two main limitations. First, by virtue, the Tafel approximation excludes the low overpotential region and therefore is inherently limited to inform on the very origin of kinetic overpotentials. It can only provide some information at higher bias. Second, it requires linear regions in the Tafel plots, which excludes bias dependent charge transfer coefficients. As a result, for rigorous application, it is limited to low-activity sp-metals. For catalysts with a higher activity, the regions of linear Tafel slopes are notoriously limited in range before the bias changes the charge transfer coefficient or mass transport limitations arise in regular three-electrode cells. These limitations can be overcome by extracting the activation energy (enthalpy) and pre-exponential factor directly from Arrhenius analysis of the steady-state HER rates at different temperatures, including at low overpotentials. According to the Eyring-equation, the pre-exponential factor in the empirical Arrhenius rate-law informs on activation entropy changes that arise from the solvent and from the surface:
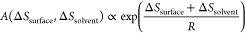
(1)

**Figure 1:**
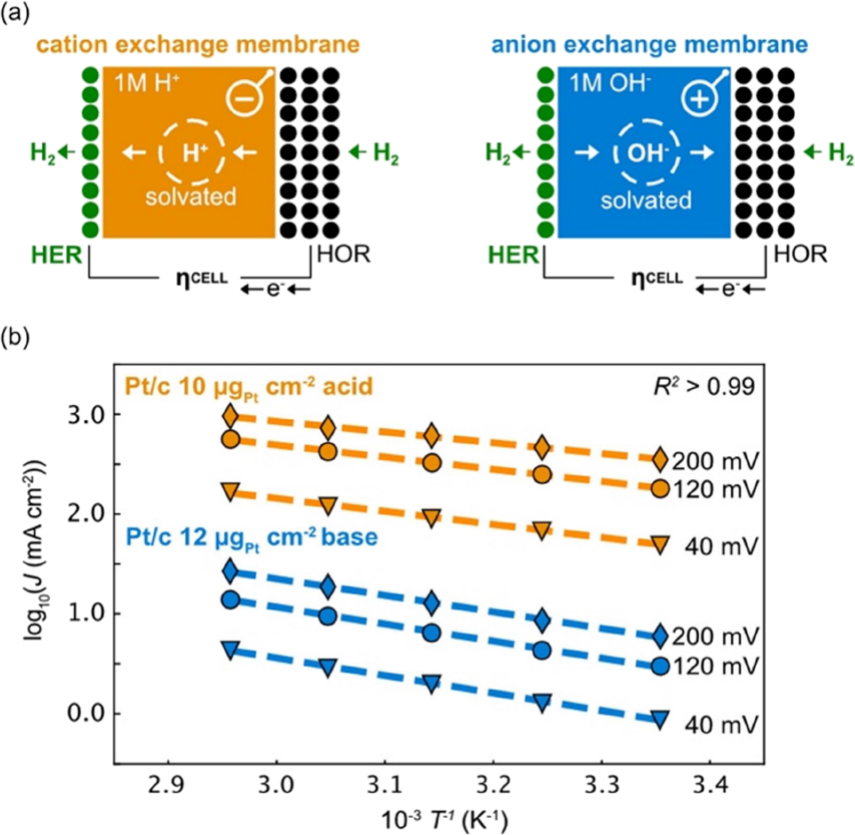
Arrhenius analysis with membrane electrode assemblies. (a) The cell potential, η_Cell_, of a H_2_^1bar^|X/C|CEM|Pt/C|H_2_^1bar^ cell with a 2 mg cm^–2^ Pt/C hydrogen oxidation reaction (HOR) reference gas diffusion electrode informs directly on hydrogen evolution reaction (HER) kinetics of 10–40 μg_metal_ cm^–2^ carbon supported catalyst X/C (X = Ir, Pt, Pd, Rh, Co, Ni, Fe, Au, Cu, Ag) (green) on the acidic cation exchange membrane (orange) or alkaline anion exchange membrane (blue). The HOR overpotential is negligible due to fast HOR kinetics and 2 orders of magnitude higher loading than the HER electrode. (b) Arrhenius analysis provides the bias dependent pre-exponential factor and activation energy. The dashed lines display the least-squares fit for three exemplary potentials with *R*^2^ > 0.99 for Pt/C.

To ensure very high mass transport, we use a hydrogen pump cell (Figure 1a) that operates the facile hydrogen oxidation reaction (HOR) reversibly on a high loading (∼2 mg cm^–2^) Pt/C gas diffusion electrode, which serves as internal reference electrode. Conversely, the total cell overpotential informs directly on the kinetics of the opposing, low loading (5–40 μg cm^–2^) HER electrode. For details about the nanoparticles, see Supplementary Table 1. Temperature-dependent kinetics are extracted by controlling the gas flow, temperature, back pressure and humidification from 25–65 °C and performing Arrhenius analysis (Figure 1b) with very high linear regression *R*^2^ > 0.98 values for almost all Arrhenius fits in this study (Supplementary Figure 1). As a result, the bias dependent changes in the activation energy, *E*_A_, and the pre-exponential factor, log *A*, surpass any variation due to statistical errors and enable us to study the bias dependent kinetics in absence and presence of real *E*_A_–log *A* compensation effects. To obtain a stable HER rate, multistep chronoamperometry steps were performed with the individual potential held between 1 and 5 min, ensuring steady-state HER currents with negligible pseudocapacitive charging or dissolution currents, except for Fe and Ni in acid that showed remaining transients even after extended potential holds (Supplementary Figures 2 and 3). These results are consistent with comparison of the extracted kinetic parameters before and after 8 h durability tests (Supplementary Figure 4). Electron microscopy of selected nanoparticles carried out before and after indicate stable performance, except for Ag/C in acid (Supplementary Figures 5–7). However, even for the latter, we observe steady HER currents (Supplementary Figures 2 and 4) when the dissolution is suppressed at high cathodic potentials. Further, we do not observe nonlinear Arrhenius behavior, e.g., due to a deformed transition-state,^25^ which could indicate tunneling or deviations from Boltzmann statistics,^26^ or other parallel processes. The pH is controlled by using an acidic cation exchange membrane with mobile H^+^ (∼1 M H^+^) and fixed anionic groups (Nafion) or an alkaline anion exchange membrane with mobile OH^–^ (∼0.1–1 M OH^–^) and fixed cationic groups (Piperion). No supporting electrolyte has been added to the gas diffusion electrodes or the pure water humified membranes, except for the experiments specifically testing for the impact of anion adsorption. For more details see Methods.

H_2_ pump cells based on membrane electrode assemblies provide superior mass transport to liquid electrolyte cells, including rotating disk electrodes. The importance of ideal mass transport was previously demonstrated when exchange current densities for Pt/C and other fast HER catalysts in acid were obtained that were orders of magnitude higher than values reported for decades based on RDE studies.^27,28^ These values have only recently been surpassed with scanning electrochemical cell microscopy^29^ that controls the exact geometry of the triple phase boundary. In summary, the ability to study fast HER kinetics in acid and the impact of hydrogen coverage (M–H) at high current densities rests on the ideal mass transport provided by the membrane electrode assembly and gas diffusion electrodes of our cells. Durable HER rates in our cells enable Arrhenius analysis with very high *R*^2^ values that are challenging to obtain in regular, liquid electrolyte cells. Recently, we used a similar setup to study bipolar membranes.^18^

### Hydrogen Evolution Reaction in Acid

The HER proceeds via a sequence of reaction steps that take place at the interface between the liquid electrolyte and a solid metal surface. In a first step, H^+^ needs to be extracted from the electrolyte by desolvating protons in acid or by dissociating water molecules (H_2_O ↔ H^+^ + OH^–^) and solvating OH^–^ ions in base. Next, during the Volmer step, a bond is formed between desolvated protons and the electrons in the metal (H^+^ + e^–^ + M → MH), which is either followed by the chemical Tafel step (MH + MH → 2 M + H_2_) or Heyrovsky step (MH + e^–^ + H^+^ → M + H_2_) that result in the formation of H_2_ gas. As such, the HER constitutes the prime example of a proton-coupled electron transfer reaction in aqueous media.

**Figure 2:**
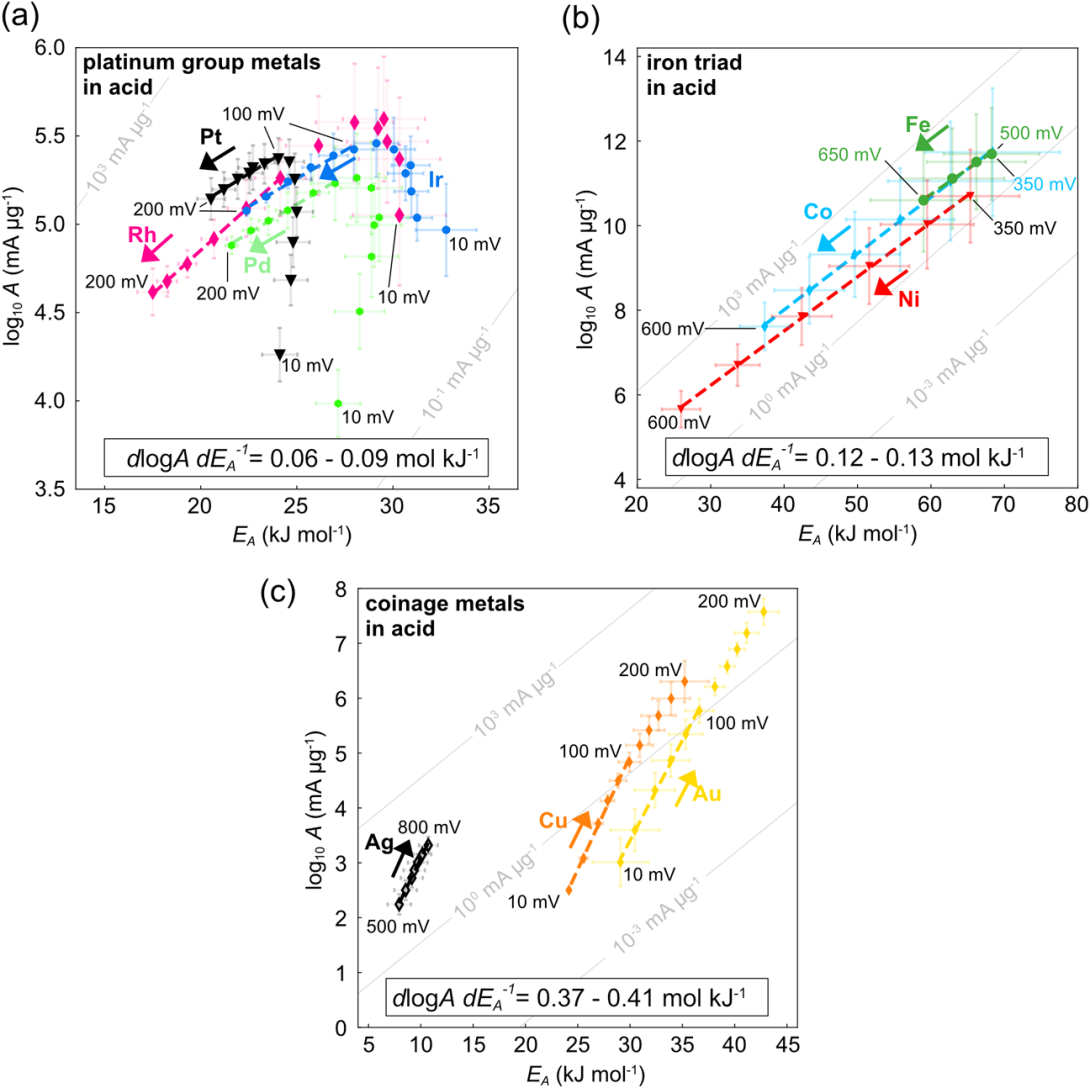
Bias dependent pre-exponential factor and activation energy for the hydrogen evolution reaction in acid. (a, b) Pre-exponential factor (log *A*) vs activation energy (*E*_A_) for the platinum group metals (PGMs) and iron triad (Fe, Co, Ni) in acid, respectively. For the iron triad only overpotentials larger (more negative) than the corrosion potentials are shown. The compensation slopes for all metals of the iron triad, Δlog *A*(η)·Δ*E*_A_(η)^−1^ ∼ 0.12–0.13 mol kJ^–1^, and for metals of the PGMs, Δlog *A*(η)·Δ*E*_A_(η)^−1^ ∼ 0.06–0.09 mol kJ^–1^, are strikingly similar within each metal group. (c) log *A* vs *E*_A_ for coinage metals in acid. In clear contrast to the d-metals, the bias increases the activation energy, but also the pre-exponential factor. The slope Δlog *A*(η)·Δ*E*_A_(η)^−1^ ∼ 0.37–0.41 mol kJ^–1^ is again identical within and distinctly different to the other groups. All listed potentials are cathodic overpotentials. For all catalysts, a low loading of 5–50 μg_metal_ cm^–2^ was used to limit the impact of mass transport and interfering kinetics of the internal reference electrode. Values are means and error bars reflect standard deviation for *E*_A_ (slope) and *A* (intercept) from Arrhenius analysis, based on five observations (temperatures). The gray diagonal lines in the background are iso-current lines at 65 °C.

Figure 2a shows the kinetic map (log *A*–*E*_A_ plot) for the PGMs (Ir, Pt, Rh, Pd) in acid. At low overpotentials, we observe a rapid rise in the apparent *A* and *E*_A_, which we assign to the impact of the reverse hydrogen oxidation rate for these fast catalysts. However, at higher bias (when the reverse rate is sufficiently suppressed) the activation energy starts to decrease and only a smaller change of the pre-exponential factor with bias is apparent, justifying the use of a Butler–Volmer/Marcus–Hush–Chidsey type formalism in acid media.^16,30,31^ This is also consistent with theoretical predictions, that the PGM d-band sufficiently overlaps with the 1s orbital of the solvated proton to directly reduce the activation enthalpy^6,12^ and speed up the initial proton (de)solvation step. In that case, the initial proton desolvation is likely inseparable from the Volmer step, which is considered the rate-determining step for the PGMs in acid.

At higher bias, we observe that increasing H coverage leads to changes of the surface configurational entropy and decreasing pre-exponential factors with bias that are compensated by activation energy changes. Previously, for the PGMs, coverage (θ) related changes in the pre-exponential factor and even activation enthalpy have been accounted for by modifying the outer-sphere rate equations with coverage terms in the pre-exponential factor.^30−32^ Conversely, in electrocatalysis the impact of coverage is generally not discussed in terms of surface configurational entropy, which has received more attention in thermal catalysis.^33,34^ However, when applying general transition state theory, these coverage terms might arise as approximation from the surface configurational entropy, depending on the coverage range. This can be seen in Supplementary Figure 8, where the surface configurational entropy can be approximated with a (2−θ) term for the (arbitrarily) chosen coverage range and (1−θ) or even less, when approaching a surface where 50% of the sites are covered. Importantly, previous approaches did not provide information on the impact of a bias dependent coverage at higher overpotentials and current densities. It was generally assumed that pH-dependent coverage terms impact the temperature-dependent exchange current density extracted in the micropolarization region^32,35^ or the electrosorption processes.^30,31^ While these important contributions could, in fact, explain the observed activity differences for the PGMs in acid and in base (more below), they do not inform on the changes at higher bias. Irrespectively, across the PGMs, we observe an entropy-enthalpy compensation slope of Δlog *A*(η)·Δ*E*_A_(η)^−1^ ∼ 0.06–0.09 mol kJ^–1^, which might arise due to the bias dependent coverage of overpotential deposited hydrogen, H_opd_, which might participate in the HER with the strongly bound underpotential deposited hydrogen, H_upd_.

The kinetic maps for the d-band iron triad (Fe, Co, Ni) in acid (Figure 2b) are shown only at higher cathodic bias, due to dissolution at lower potentials. For the Arrhenius analysis, reliable *R*^2^ are obtained (Supplementary Figure 1) by conditioning the cells at large negative potentials and then subsequently stepping down to lower cathodic overpotentials until apparent catalyst dissolution. This yields steady state HER rates for Co and Ni, whereas Fe exhibits remaining transient currents during the potential holds (Supplementary Figure 2). Similarly to the PGMs, the bias reduces the activation energy directly for the iron triad in acid, likely due to the sufficient overlap of the 3d band with the 1s orbital of the proton, which was predicted theoretically before.^12^ However, the decreasing activation energy is compensated by a pre-exponential factor that falls by over 5 orders of magnitude with bias (Figure 2b), which might indicate large surface configurational entropy changes (Supplementary Figure 8). This is a rather surprising finding, since the strongly bound H_upd_ is already present at electrochemical equilibrium and generally considered the primary HER intermediate on these metals. Based on DFT calculations on single crystal surfaces,^15^ the iron triad is expected to follow the Sabatier principle, where the role of the bias is then to simply reduce the free energy of this primary H_upd_ intermediate. However, polycrystalline nanoparticles might provide a great number of sites that can increase their H_opd_ coverage from almost zero to intermediate coverages, which could lead to a coparticipation of H_opd_ with H_upd_ in the HER, and possibly the observed compensation effect. Simultaneously, the steady-state hydrogen coverage could be reduced when approaching the onset potentials of dissolution, impacting this surface configurational entropy contribution. Across the iron triad, we find an almost identical compensation slope of Δlog *A*(η)·Δ*E*_A_(η)^−1^ ∼ 0.12–0.13 mol kJ^–1^, which is substantially larger than the one for the PGMs. The different slopes might be a reflection of the Heyrovsky rds for the iron triad in acid,^12^ compared to the Volmer step for the PGMs. In the literature this distinction is typically based on different Tafel slopes extracted at a constant temperature and assuming a constant pre-exponential factor. Noteworthy, at a constant bias of 600 mV, we find a correlation of log *A* and *E*_A_ with the atomic number for Fe, Co, and Ni. Finally, we caution of overinterpretation of our Fe data in acid, since this is the least stable catalyst in this study and which showed pronounced current transients even after extended potential holds.

The coinage metals in acid contrast the behavior of the d metals altogether (Figure 2c). The rates increase due to increasing pre-exponential factors and despite increasing activation energies, starkly contrasting Butler–Volmer behavior. Importantly, no mobile electrolyte ions were present for the data in Figure 2c. This strongly suggests that the bias dependent hydrogen bond network is a fundamentally important component for electrocatalyst kinetics in aqueous solvents (more below). For Ag, we only show higher overpotentials, due to unstable performance and unreliable Arrhenius analysis at lower overpotentials (Supplementary Figure 2). In contrast to the rising pre-exponential factors for fast PGMs in acid, the reverse hydrogen oxidation rates are negligible for the coinage metals and cannot explain these trends. Furthermore, molecular dynamics (MD) predict increasing activation energies with bias,^7,36^ due to increasing excess charge and water dipole ordering. However, as outlined above, entropic changes are theoretically not been addressed satisfyingly.

Increasing pre-exponential factors with bias can be reconciled by entropic changes in the interfacial solvent, as argued previously by Conway.^21−24^ However, for the HER kinetics on the sp-metals, the activation energy appears to always decrease with bias, whereas the activation entropy increases or decreases. In contrast, we observe increasing activation energies with bias across the coinage metals in acid and previously for a whole range of reactions and catalysts in base and under other conditions.^18,19^ We hypothesize, that for these conditions, the bias induces interfacial excess charge which increasingly orders and connects the hydrogen-bond network. In fact, recent results by Chen and co-workers^37^ based on extensive MD simulations not only indicate the presence of an interfacial “shuttle” water molecule in acid and in base, which was also observed previously,^7^ but, importantly, a bias dependent interfacial hydrogen bond connectivity. Conversely, with increasing connectivity in an iso-energetic hydrogen bond network, the OH^–^ and H^+^ could get increasingly delocalized, which would increase the transition state entropy. Additionally, the bias might also decrease the entropy of the H_2_O reactant molecule at the initial state, *S*_i_. In both cases, the activation entropy, Δ*S* = *S*_t_ – *S*_i_, increases. However, in all cases, increasing the hydrogen bond ordering increases the activation enthalpy. Clearly, more powerful MD simulations and operando spectroscopy will be critical to test this qualitive description and obtain molecular level insights into the entropic and enthalpic processes across the double layer. Irrespectively, our previous and new results strongly point toward entropic and enthalpic changes in the solvent that cause the constant compensation slope (Δlog *A*(η)·Δ*E*_A_(η)^−1^ ∼ 0.37–0.41 mol kJ^–1^) across the coinage metals in acid (and similarly in base). Noteworthy, studies employing Ag and Au foils in liquid acidic electrolytes obtained similar slopes at low bias,^38,39^ but observed a switch in the kinetic regime at higher bias (more below).

The absolute values of log *A* and *E*_A_ at a constant potential or current density across all tested metals and conditions might be impacted by the exact chemical composition and atomic catalyst structure, which can furthermore impact the surface coverage and electric field distribution inside the electrochemical double layer. Here, we focus on the constant slopes for the bias dependence in log *A* and *E*_A_. To reconcile these broad trends solely with (material specific) chemical compositional or structural changes would require us to postulate general chemical and structural changes that are shared among all catalysts in one metal group and that impact the HER kinetics in the exact same way. In particular, to reconcile increasing activation energies and pre-exponential factors with bias (e.g., Figure 2c) with changes on the catalyst surface alone would require a decreasing coverage of the primary intermediate with increasing rates. To that end, some previous studies proposed parallel surface reactions^40^ which are however specific to the surface chemistry and cannot explain our results across many different reactions^18,19^ and catalysts. Further, our results in alkali, the capacitance and effects of anions below further indicate a key role of the activation entropy changes in the solvent. We note that the steady-state currents used to extract the kinetics for the coinage metals, show negligible transient double-layer charging or dissolution. This is also supported by robust Arrhenius analysis, even after an 8 h durability test for Au (Supplementary Figure 4b).

### Hydrogen Evolution Reaction in Alkali

**Figure 3:**
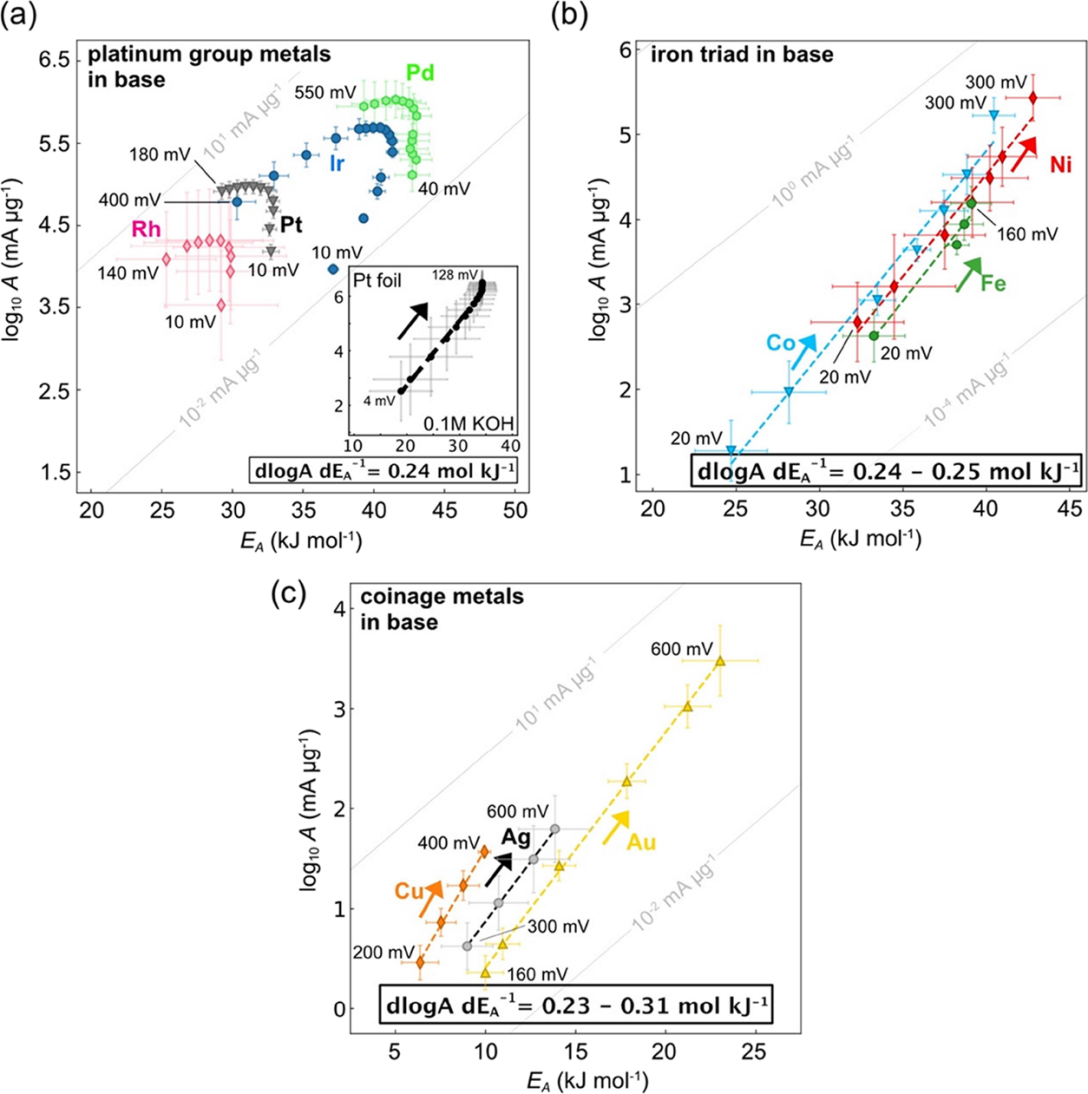
Bias dependent pre-exponential factor and activation energy for the hydrogen evolution reaction in alkali. (a, b) log *A* vs *E*_A_ for PGMs and iron triad in alkali, respectively. The 3–4 nm PGM nanoparticles display a similar behavior as in acid, but with a higher *E*_A_(η). Reference measurements on polycrystalline Pt foils in 0.1 M KOH (inset in (a)) result in the same compensation slope as for the other metals in base. (c) log *A* vs *E*_A_ for coinage metals in alkali with a very similar compensation slope as the d-band metals in alkali (panel (b)), but substantially lower compared to the slopes of the coinage metals in acid of Δlog *A*(η)·Δ*E*_A_(η)^−1^ ∼ 0.37–0.41 mol kJ^–1^ (Figure 2c). All listed potentials are cathodic overpotentials. For all catalysts, a low loading of 5–50 μg_metal_ cm^–2^ was used to limit the impact of mass transport and interfering kinetics of the internal reference electrode. Values are means and error bars reflect standard deviation for *E*_A_ (slope) and *A* (intercept) from Arrhenius analysis, based on five observations (temperatures). The gray diagonal lines in the background are iso-current lines at 65 °C, i.e., pairs of log *A* and *E*_A_ that result in the same current.

In alkali (Figure 3a–c), we find that for all catalysts, except the nanoparticle PGMs, increasing rates coincide with increasing activation energies and pre-exponential factors. We observe almost identical compensation slopes across these chemically dissimilar metals (*m*_b_ = Δlog *A*(η)·Δ*E*_A_(η)^−1^ ∼ 0.23–0.25 mol kJ^–1^), which are very similar to the slopes we observed previously^18,19^ when studying water dissociation and hydroxide solvation kinetics in polymeric bipolar membranes and at polycrystalline metal–KOH interfaces (∼0.24–0.25 mol kJ^–1^). The only exception is Cu, which shows a slightly higher slope of around Δlog *A*(η)·Δ*E*_A_(η)^−1^ ∼ 0.31. Importantly, all observed slopes in alkali are distinctly lower than for the coinage metals in acid (*m*_a_ = Δlog *A*(η)·Δ*E*_A_(η)^−1^ ∼ 0.37–0.41 mol kJ^–1^). In line with our previous hypothesis,^18^ our results now show that the difference between H_3_O^+^ (de)solvation *vs.* water dissociation and OH^–^ solvation can manifest itself as different compensation slopes in acid and in base for some of the metals. To increase the activation entropy for hydroxide solvation, more excess charge is needed than for proton solvation, which is penalized by a larger incremental increase of the activation energy per increment of activation entropy. The slope ratio, *m*_a_*m*_b_^–1^ ∼ 1.3–1.6, might be related to the behavior of H_3_O^+^ and OH^–^ in bulk water,^41,42^ but impacted by the ordered transition state.

The PGM nanoparticles show a higher *E*_A_ in alkali (Figure 3a) than in acid (Figure 2a) and follow approximately Butler–Volmer type kinetics, whereas the exact absolute values of *E*_A_ and *A* depend on the catalyst and loading (Supplementary Figure 9). As mentioned above, such activity differences could be explained by pH-dependent and competing coverage terms of H_upd_, H_opd_ and, potentially, OH_ad_, that are already present in micropolarization or electrosorption regions^30−32^ and, thus, might impact the activation enthalpy at low overpotentials. In contrast to acid, at a constant low overpotential, the absolute value of the pre-exponential factor and activation energy compensate between the different catalysts in base, which could be related to the impact of OH_ad_ coverage depending on the surface chemistry and, in fact, work function and potential of zero charge (more below). At higher bias, we observe again entropy–enthalpy compensation slopes that are likely related to the entropic and enthalpic impact of an increasing coverage of the primary H_opd_ and interaction with the H_upd_. Finally, when we obtain *E*_A_ and *A* for a polycrystalline Pt foil in 0.1 M KOH (inset Figure 3a), we observe the same slope, Δlog *A*(η)·Δ*E*_A_(η)^−1^ ∼ 0.24 mol kJ^–1^, as for other catalysts in alkali and in line with our recent results.^19^ This suggests that the exact atomic structure of the nanoparticles might impact the electric field profiles inside the double layer. Hot spots at undercoordinated sites of ∼1–3 nm PGM nanoparticles (Supplementary Table 1) might boost the pre-exponential factor compared to molecularly flat surfaces. Due to the discussed mass-transport limitations for HER kinetics in acid, we are unable to study the same polycrystalline Pt foil in liquid acid. Irrespectively, studying structure–electric field relationships will be important in the future.

Ion exchange during the pretreatment of the anion exchange ionomer and membrane might impact the HER kinetics in alkali. Whereas the commercial Nafion ionomer and membrane are provided in protonated form, the PiperIon anion exchange polymer requires an additional pretreatment to exchange bicarbonate ions with hydroxide ions. Therefore, we tested for the impact of remaining bicarbonate ions in our experiments, by performing a complete set of experiments with gas diffusion electrodes in their bicarbonate form. In this case, remaining bicarbonate ions in the GDE are displaced with hydroxide ions generated during the conditioning inside the MEA (H_2_O + 2e^–^ → H_2_ + 2OH^–^). Supplementary Figure 10 shows very similar kinetics as in Figure 3, further supporting a fundamental role of the interfacial water and hydrogen coverage, even in absence of electrolyte ion effects that are likely to modify the kinetics.

Our data and interpretation are consistent with pH-dependent electrosorption studies on Pt(111) single crystals^31^ that ruled out surface configurational entropy changes^43^ and instead proposed pH-dependent water ordering effects to explain non-Nernstian HER kinetics with pH. In fact, pH dependent (de)protonation rates across the whole pH range of graphite-conjugated carboxylic acids (GC-COOH ↔ GC–COO^–^ + H^+^) can be recovered by combining one fast rate constant in acid (related to proton solvation) and a slow rate in base (related to hydroxide solvation) in a total rate expression.^30^ Similar conclusions were drawn when the rate-limiting charge transfer step on Pd was studied in isolation, which involves water dissociation and hydroxide solvation in alkali (2H_2_O + 2e^–^ → H_2_ + 2OH^–^).^44^ Laser-induced temperature jump measurements^45−47^ showed that the surface charge increases in alkali compared to acid on Pt. This was related to the difference in the potential of zero total charge and the onset potential for Faradaic current. Conversely, the notion developed in the literature that a more ordered hydrogen bond network in alkali than in acid is more difficult to reorganize during ion (de)solvation and, thus, results in larger energy barriers.^45^ However, these studies where limited to low current densities and did not inform on the bias dependent activation enthalpy or entropy.

### Absence of Unifying Kinetics across Different Metals

**Figure 4:**
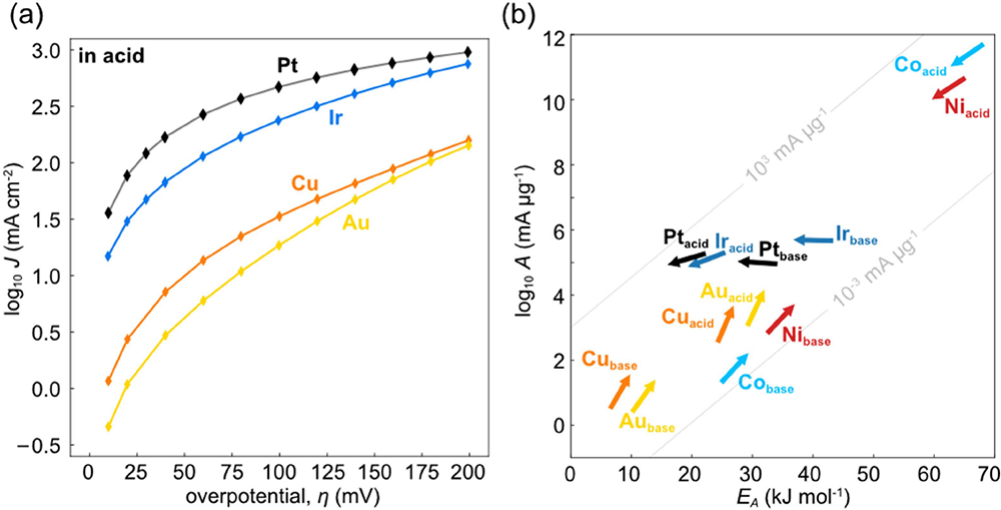
The absence of unifying HER kinetics. (a) Exemplary Tafel plots for the HER in acid at 65 °C. (b) Overview of log *A* vs *E*_A_ across metals from Figure 2 and 3 at low overpotentials with arrow direction based on two successive potentials, for the PGMs a slightly higher potential was chosen due to the impact of the reverse hydrogen oxidation reaction. Gray diagonal lines in the background are iso-current lines at 65 °C.

Figure 4a shows Tafel (log *j* vs η) plots for a range of HER catalysts in acid with seemingly linear regions at higher bias. Linear Tafel slopes are often interpreted as reflection of Butler–Volmer type kinetics and how the bias reduces the activation energy, including to extract the number of electrons involved in the reaction. The pre-exponential factor is constant in these pictures or primarily dependent on the intermediate coverage. However, even in absence of mass transport limitations, a linear Tafel slope at a single temperature is insufficient to invoke Butler–Volmer kinetics. For example, the Tafel slopes of Au, Cu, and Pt all display linear regions (Figure 4a), yet, the HER kinetics are diametrically different (Figure 2a–c), due to compensation effects between *E*_A_ and log *A*. Temperature dependent studies are always needed to inform on the accurate partitioning of Δ*G*(η) into Δ*S*(η) and Δ*H*(η). Temperature dependent Tafel slopes inform on the bias susceptibility of the entropy (α_S_) and enthalpy (α_H_) separately, as shown by Conway,^21−24^ albeit with limitations for bias dependent charge transfer coefficients for non-sp-metals, as discussed above.

At low overpotentials, the HER kinetics of the different catalysts in Figure 4b show a trend that is very similar to the ones observed by Zeradjanin and co-workers when studying the temperature dependence of the exchange current density across a large set of polycrystalline metal surfaces in acid and in base.^48,49^ HER activity, as quantified by the exchange current density extrapolated from high overpotentials to equilibrium or extracted in the micropolarization region, appears to correlate only broadly with the work function (and related potential of zero charge). Prominently, Trasatti argued that the work function—HER activity correlation for the coinage and sp metals should form a different group than for the PGMs and iron triade,^50,51^ although later results questioned those findings.^52^ Recently, Zeradjanin and co-workers showed that the number of outer electrons in the d-band appears to correlate with log A and E_A_ at a low current density (the coinage metals formed a distinctly different group).^53^

Recently, we observed a similar compensation at low overpotentials for metal oxide catalysts inside bipolar membranes^18^ and argued that the pH dependent coverage of acid–base sites, depending on the oxide’s point of zero charge and, thus, work function, fundamentally links surface charge and surface configurational entropy at low bias. At electrochemical equilibrium, the local chemical potential differences are fully compensated by electrostatic potential differences, which links coverage changes with the surface charge and, thus, the metal’s work function^50,51^ or d-band level.^53^ While this might explain the compensation across different catalysts at low bias or current density (Figure 4b), we note that the compensation at low bias by in and of itself is rather unsurprising, given the large activation energy differences, that are determined in the experimental temperature range with very high *R*^2^ linear regression values (Supplementary Figure 1). A constant pre-exponential factor would lead to rates that differ by about ∼10^10^ between the coinage metals in base (∼10 kj mol^–1^) and the iron triad in acid (∼70 kj mol^–1^) due to strong impact of the Boltzmann factor (exp(−*E*_a_/*k*_B_*T*)). This is often neglected when overemphasizing activation energies over Arrhenius pre-exponential factors. Irrespectively, the HER kinetics at low bias are is insufficient to explain what happens if uncompensated charge emerges under bias.

HER catalysts can be grouped into at least three categories according to their bias dependent log *A* and *E*_A_, in line with predictions on theoretical grounds by Schmickler and co-workers^12^; the PGMs, iron triad and coinage metals. Due to safety concerns, we did not include sp-metal nanoparticles in our study, which likely form a separate group, with decreasing *E*_A_ and increasing or decreasing *A*.^21^ Under bias, the local chemical potentials of electrons and ions can change, depending on the metal, which entails uncompensated charge and net electric fields. Under these conditions, the activation entropy changes between the surface and the solvent start to deviate, i.e., the catalysts in Figure 4b move away from the common compensation slope at low overpotentials. The approximately nonpolarizable and reversible PGMs are exceptional in that they move almost horizontally to decreasing activation energies. In contrast, the coinage metals deviate with bias not by much from the compensation slope across catalysts at low bias, which further implies a surface charge related origin for the compensation slope at low bias.

The pH dependent HER kinetics can manifest themselves as larger activation energies in base than in acid (PGMs), as commonly assumed, or by a different bias dependence, i.e., the compensation slopes with increasing log *A*(η) and *E*_A_(η) are absent in acid and present in base (iron triad) or different between acid and base (coinage metals). These findings go substantially beyond prevailing pictures that try to explain the pH dependent HER kinetics.

### Role of Bias Dependent Capacitance

The kinetic maps in Figures 2–4 imply a distinctly different impact of the bias on the H surface coverage and interfacial dipole ordering inside the electrochemical double layer, depending on the metal group and electrolyte pH. Previously, we correlated changes in *E*_A_ and *A* directly to changes in the space charge (pseudo)capacitance of bipolar membrane junctions.^18^ There, the pseudocapacitive deprotonation and dehydroxylation of fixed ion-exchange groups leads to bias dependent excess charge that entails electric fields that induces entropic changes in the interfacial water layer. Conversely, we hypothesized that the different behavior of the three metal groups in Figures 2 and 3 is reflected in their capacitance, especially for the coinage metals. Thus, we studied the bias dependent impedance (Supplementary Figures 11–20). For details, about the data analysis, see Supplementary Note 1.

Due to the use of pure-water humidified membranes, specific ion adsorption is negligible in Supplementary Figures 19 and 20, simplifying the interpretation of the capacitance. The absolute capacitance depends on the exact (nanoparticle) catalyst structure and chemical composition. Furthermore, it can be impacted by the reduction of surface oxides. Despite this lack of resolution, our results indicate that the bias dependent capacitive behavior of all of these metals is similar in magnitude within a given group of related materials and distinctly different between the metal groups in acid and alkali. Moreover, these results also mirror the grouping in the bias dependent kinetic maps in Figures 2 and 3. This finding is important to understand the fundamental origin of the compensation slopes in acid and base. With the exception of Cu in base (below) higher pseudocapacitive changes occur in acid for d-band metal catalysts that directly reduce the activation enthalpy and change the coverage of hydrogen on the surface, which leads to a (surface configurational) compensating decrease in the pre-exponential factor. In contrast, for all metals in alkali and the coinage metals in acid (except for Cu), the capacitance changes are lower and of similar magnitude, and likely governed by water dipole reorientation^36^ also observed via X-ray absorption^54^ and vibrational sum frequency spectroscopy^55^ and likely similar to sp-metals.^56^

When we compare the coinage metals, that show entropy–enthalpy compensation effects in acid and in base, we observe that the changes in the pre-exponential factor rise faster but also saturate earlier with capacitance changes. This might be related to a different electric field response^42^ of hydroxide–water complexes in alkali,^57,58^ compared to proton–water complexes in acid.^59−61^ However, the Cu nanoparticles appear to be an exception when studied in hydroxide pre-exchanged GDEs. For these, the capacitance changes are substantially larger, potentially due to the impact of the reduction of surface oxides that formed during extended hydroxide exchange or dissolution. Similarly, for Ni the capacitance also increases after extended exposure to 1 M KOH but substantially less than for Cu. Irrespectively, the capacitance data collectively conflicts with pictures that try to reconcile slower HER kinetics only with competing OH-coverage and/or surface configurational entropy changes that are specific to the surface chemistry and would likely lead to capacitance variations that are of similar magnitude between acid and base. For more discussion, including for the iron triad and PGMs, see Supplementary Note 1 and Supplementary Figures 19, 20.

### Role of Specific Anion Adsorption

Intrinsic water ordering and H surface coverage effects likely have a primary effect in our results in Figures 1–4, since all MEA components are rinsed with copious amounts of pure water before assembly and only fed with pure water humidified gases during operation. Noteworthy, the compensation slopes in the alkaline MEA (Figure 3b,c) that is fed with pure water are very similar to the slopes we measured recently for polycrystalline metal foils in liquid KOH.^18^ This suggests that there is a fundamental, electrolyte ion independent role of the interfacial hydrogen bond network in the catalyst kinetics. However, when switching to liquid base, some cations might modify the transition state of the HER.^62−65^

In acidic Nafion, the compensation slopes for the coinage metals (Figure 2c) are similar to the ones observed by others at low overpotentials in liquid acid for single and polycrystalline Ag and Au metal foils.^38,39^ However, at higher overpotentials, these studies observed a saturating pre-exponential factor and a decreasing activation energy, i.e., Butler–Volmer kinetics. This was related to specific anion adsorption,^66^ which can be favorable even on cathodic electrodes depending on the equilibrium free energy profile and applied bias range.^67,68^ Therefore, we tested whether anion adsorption induces a switch from the slow compensation regime to the fast Butler–Volmer regime, by immersing the Nafion ionomer containing GDEs for 12 h in 1 M HClO_4_, H_2_SO_4_, and KCL before assembly (details in Methods). Nafion nominally only contains pure water, mobile protons and fixed sulfonate groups. However, for high (>∼0.1 M) electrolyte concentrations the ion selectivity of the ion exchange polymers falls substantially,^69,70^ leading to pronounced co-ion transport of mobile electrolyte anions, detrimentally impacting, e.g., the product purity in electrodialysis. However, here, this feature enables us to expose the nanoparticles to anions (ClO_4_^–^, Cl^–^, SO_4_^–^) at a high concentration (0.1–1 M) at equilibrium. After assembly and during operation, the concentration in the GDE will be substantially reduced, since solvated anions in the bulk of the GDE should be repelled from the negative electrode and distribute over the MEA, reestablishing the cation-selectivity of the Nafion ionomer in the GDE. This complicates accurate quantification of the electrolyte ion concentrations in the complex membrane-ionomer-catalyst environment. However, here, we only test whether the mere presence of anions impacts the kinetics compared to the pure water humidified MEAs.

**Figure 5:**
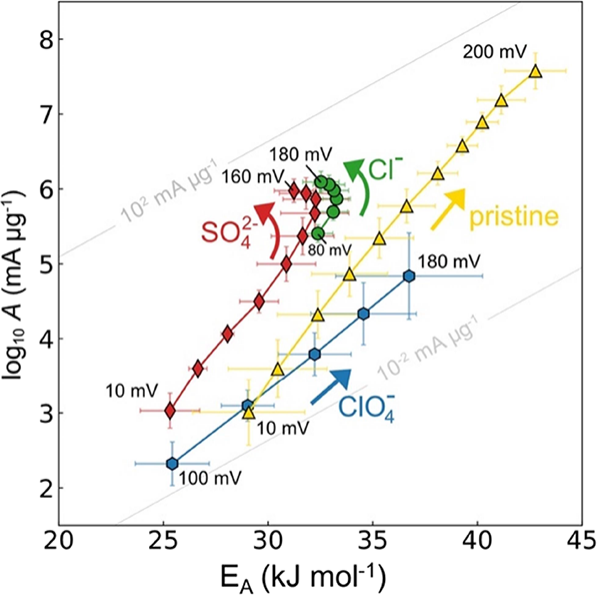
Impact of anion adsorption on the HER kinetics of Au nanoparticles. The Nafion-containing gas diffusion electrodes were used as prepared (pristine) or soaked for 12 h in 1 M HClO_4_, H_2_SO_4_, and KCl prior to assembly. Subsequently, the GDEs were operated in the H_2_ pump membrane electrode assembly that uses the reversible hydrogen oxidation reaction as counter and internal reference electrode. Strongly adsorbing anions (SO_4_^2–^ and Cl^–^) lead to a distinct change in the kinetics. At higher overpotentials, the pre-exponential factor saturates and the kinetics switch into a fast Butler–Volmer regime, where the reduction of the activation energy in the Boltzmann factor leads to rapid acceleration of the rates. In contrast, weakly adsorbing anions (ClO_4_^–^) lead to negligible change in the bias dependent kinetics compared to the pristine Nafion. Note, the absolute rate is substantially suppressed for ClO_4_^–^, which might be caused by nontrivial (co)ion-exchange. Bias dependent pre-exponential factor, log *A*(η), and activation energy, *E*_A_(η), from Arrhenius analysis, based on five observations (temperatures). The gray diagonal lines in the background are iso-current lines at 65 °C, i.e., pairs of log *A* and *E*_A_ that result in the same current.

Figure 5 shows the kinetic map for the Au nanoparticles operated in pure water humidified Nafion (Figure 2c), and exposed to different anion containing electrolytes. Clearly, the weakly absorbing ClO_4_^–^ leads to negligible impact on the bias dependent kinetics (but suppression of the absolute rates compared to pristine Nafion), whereas the strongly absorbing Cl^–^ and SO_4_^–^ impact the kinetics in a striking way—the pre-exponential factor saturates at higher bias and the kinetics switch to a fast Butler–Volmer regime. This transition clearly suggests that anions can indeed help speed up the initial solvation step by providing a beneficial electrostatic environment which might stabilize the excess charge density and help to extract solvated protons from the solution.^66^ Importantly, in contrast to previous hypotheses,^66^ the compensation at low bias is not caused (but can be modified) by the presence of anions, providing further support for the fundamental role of the interfacial hydrogen bond network. Whereas GDE exposure to H_2_SO_4_ does not induce any cations other than protons and, thus, supports the importance of SO_4_^–^ adsorption, in the case of KCL, the K^+^ might additionally modify the electrostatic environment, depending on the degree of solvation and concentration at the interface.^37^ Clearly more controlled in-depth studies are needed to parse out the intricate impact of electrolyte ions on the electrocatalyst kinetics.

The impact of anion adsorption on the HER kinetics of the coinage metals is key to understand the product selectivity between the HER and CO_2_ reduction reaction (CO_2_RR) on the same catalyst surfaces. In acid, the HER generally outcompetes the CO_2_RR unless concentration polarization is induced at higher bias and current densities.^71,72^Figure 5 suggests that the HER activity on coinage metals is strongly influenced by specific anion adsorption which induces fast Butler–Volmer kinetics. During concentration polarization and a locally increasing pH in liquid electrolytes, the charge balancing anion concentration might decrease at the interface, too. According to Figure 5, this would suppress HER activity, because it promotes entropy–enthalpy compensation over (adiabatic) Butler–Volmer kinetics. In other words, the product selectivity might be tunable by promoting or suppressing fast Butler–Volmer type kinetics over slow compensation effects, depending on the desired product. Finally, beyond fundamental interest, the results in Figure 5 are important when comparing catalyst activity differences between liquid electrolyte cells and pure water-operated membrane fuel cells and water electrolyzers. In fact, the addition of anions (or cations) to the pure water systems might accelerate the kinetics.

## Discussion

Current electrocatalysis theory and education rests on outer-sphere Butler–Volmer/Marcus–Hush type behavior of few fast PGM-based HER catalysts in acid.^12^ For these highly reversible and nonpolarizable catalysts, the bias primarily reduces (increases) the activation enthalpy of the forward (reverse) rates. Conversely, the effect of the solvent is limited to solvent reorganization terms in the activation enthalpy.^73^ These narrow conditions support the use of thermodynamic activity descriptors, such as the hydrogen binding energy, and conventional DFT to estimate the catalyst activity.^1^ However, as our results show, the majority of catalysts do not display simple outer-sphere kinetics in acid, let alone in base, and, instead, build-up substantial excess charge at the surface. These results are obtained by performing statistically robust, bias dependent Arrhenius analysis with very high linear regression *R*^2^ values (>0.99) and changes in log *A* and *E*_A_ that exceed any statistical variation. Such conditions cannot be captured accurately by DFT due to the limitation of the charge neutral unit cell, but possibly require combination with advanced MD^36,37,74^ and machine learning potentials.^4^ Finally, the absence of unifying bias dependent kinetics casts substantial doubt on the validity of applying the Sabatier principle and volcano activity plots across diametrically different materials, and especially any claims that the catalyst activity can be reduced to the hydrogen adsorption energy.^1^ Our conclusions resonate with experimental studies by Zeradjanin and co-workers^48,49,52,75^ and considerations by Schmickler and Trasatti that concluded that these oversimplified models misrepresented catalyst activity right from the start.^12,13^

**Figure 6:**
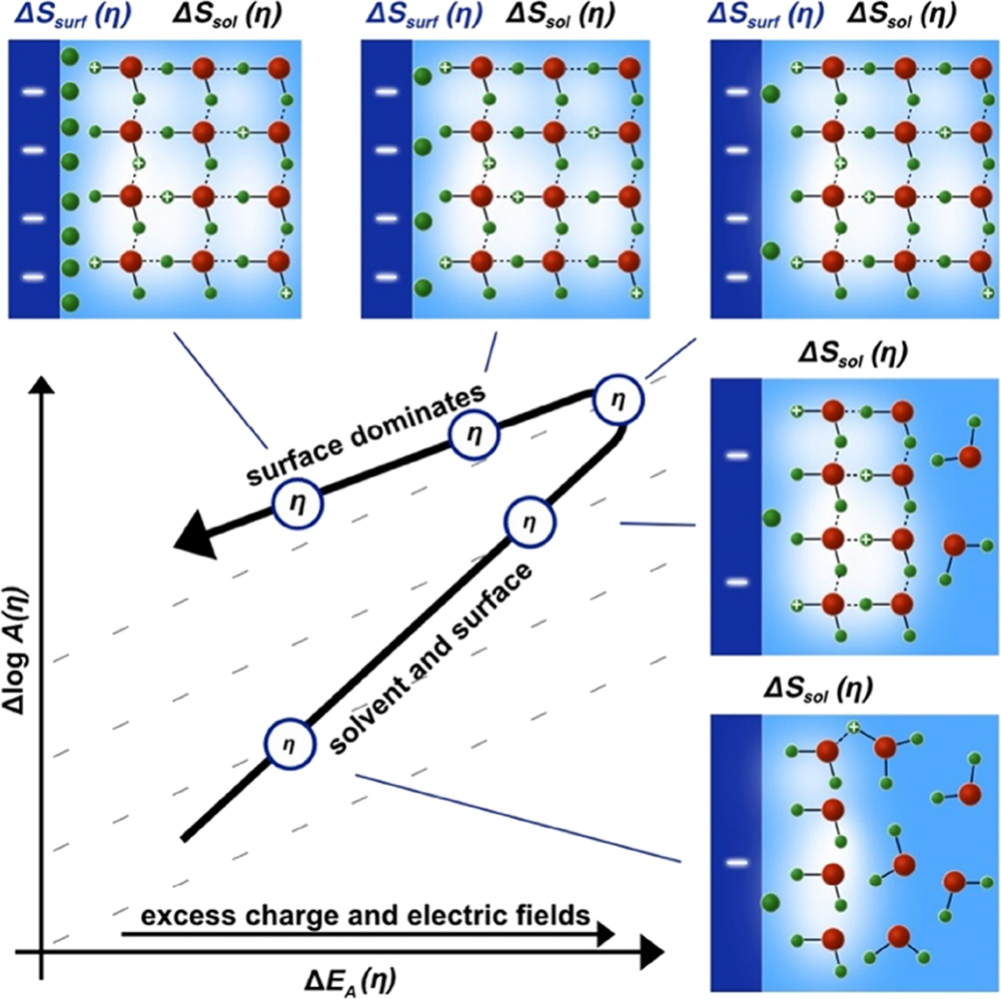
Bias dependent activation enthalpy and entropy compensation in electrocatalysis. Starting at low overpotentials, the excess charge and, thus, electric fields, at the solid–electrolyte interface increase, causing an increasing activation entropy for the solvation step, Δ*S*_sol_(η), likely due to ion delocalization in the ordered hydrogen bond network. However, the increasing excess charge at the surface is penalized by an increasing activation energy, *E*_A_(η). Once the water is ordered sufficiently, ion solvation becomes fast and the bias starts to reduce the activation enthalpy of the intermediates over the (capacitively charged) catalyst surface. This transition region in the kinetics is critical to speed up reactions and switch into the fast Butler–Volmer regime where activation energies decrease with bias. However, with increasing coverage of intermediates (green circles), the surface configurational entropy, Δ*S*_surf_(η), decreases due to a change of the number of microstates on the surface. Here, different compensation slopes might reflect what are traditionally considered the Volmer or Heyrovsky rate-determining step.

The bias dependent activation entropy in the pre-exponential factor deserves as much attention as the activation energy, even for a (seemingly) simple reaction, such as the HER. As such, our results demonstrate that the “Conway-picture” of electrocatalysis is valid for a much broader range of catalysts and conditions and by no means limited to acidic proton donors at the ideally polarizable Hg electrode.^21−24^ The prominence of the mercury drop electrode has historically likely led to a scarcity of temperature dependent data sets in alkali. Thus, by combining our previous results in alkali,^18,19^ the new results presented here and prior ones from the literature,^37,66^ we infer the following tentative picture on a general bias dependence in electrocatalysis. With increasing polarization, excess charge and electric fields lead to increasing ordering of the interfacial hydrogen bond network (Figure 6). This (entropically) boosts the pre-exponential factor, because the ion is delocalized over an increasingly ordered and connected hydrogen bond network,^37^ which increases the (configurational) entropy of the transition state, *S*_t_. In other words, the number of accessible microstates increases in the transition state, which increases the probability of the barrier crossing event. Additionally, the entropy of the H_2_O reactant state, *S*_i_, might also be reduced, which would further increase the activation entropy, Δ*S* = *S*_t_ – *S*_i_. However, the increasing ordering of the hydrogen bond network comes at the (enthalpic) expense of increasing activation energies. For larger hydroxide ions, the delocalization might be lower than for protons, potentially causing the pH-dependent compensation slopes and HER kinetics. Platinum group metals with their larger d-band overlap with the 1s orbital of the proton bypass this separate solvation step and reduce the activation enthalpy directly.

Once the activation entropy of the solvation transition state is large enough, the initial (de)solvation step becomes fast. The kinetics switch from a slow regime impacted by interfacial solvation to a fast one that is dominated by the surface energetics. At this point, the bias does not induce more excess charge at the surface, but it starts to reduce the activation enthalpy over the capacitively charged surface. Conversely, the bias dependent kinetics start to be governed by adiabatic electron transfer, resulting in Marcus–Hush/Butler–Volmer-type behavior. As we have shown here, this transition region can be impacted by electrolyte ions that might aid the preorganization of the interfacial hydrogen bond network and/or draw protons closer to the surface to enable overlap of the metal orbital with the 1s orbital of the proton.^66^ However, even with decreasing activation energies with bias, the coverage of surface intermediates can induce changes in surface configurational entropy and related compensation effects, as is known from thermal catalysis. Here, the impact of the bias on the hydrogen binding energy becomes an important factor and the different compensation slopes for the different metal groups might reflect the Heyrovsky vs Volmer rate-determining steps.

This transition in the kinetics from a regime limited by interfacial (de)solvation to one controlled by surface energetics and the two opposing compensation effects, i.e., one where the *E*_A_ and log *A* increase and one where *E*_A_ and log *A* decrease with increasing bias must have evaded Conway.^21^ For the sp-metals, he appears to have never observed a transition region and only reported that *E*_A_ always decreased with bias, whereas log *A* decreased or increased. Irrespectively, from a traditional catalysis view, this transition region in the kinetics could be considered as being the point where the “pre-step” becomes fast and the catalyst starts to act according to heterogeneous catalysis working principles. However, interfacial solvation is an activated process that adheres to a general framework based on transition state theory. It is *not* a simple mass transport limitation. In polar solvents, interfacial solvation is as much part of the interface, as reactions on the surface. This is critical to understand a whole breadth of electrochemistry more comprehensively. Every single corrosion, electrodeposition, oxidation or ion intercalation step, as well as proton conduction across proton-pumps and motors require ionic charge transfer of solvated ions in polar solvents—whether the reaction involves net electron transfer or not. As such, interfacial solvation constitutes the first step that separates outer-sphere from inner-sphere electrochemistry at heterogeneous interfaces. Fully appreciating this is key to update and broaden our education and to develop new (bio)electrochemical technology, such as when exposing heterogeneous Bronsted acid catalysts to strong electrochemical potential gradients.^76^

Electric fields might also change the vibrational entropy (the transmission coefficient or frequency) or lead to related resonance effects.^77,78^ Clearly, in absence of detailed spectroscopic data and theoretical models, the schematic in Figure 6 can only serve as tentative conceptual picture. In fact, we expect that our picture will be subject to future modifications. In particular, we assume that structural changes of the catalysts under bias can impact the compensation slopes, as they might modify the electric field distribution inside the double layer. Further, while our study is based on Arrhenius analysis of steady state HER currents, the respective transition state might be impacted by parallel catalyst dissolution. Additionally, the exact nature of the excess charge at the interface, including the response of the local structural and chemical environment, is likely critical to understand the transition region between the solvation “pre-step” and the active solid structure. To address entropic and enthalpic changes across electrocatalysis, a great effort between updated theory,^3,4,36,79^ operando spectroscopy^37,80^ and microscopy and large expansion of temperature dependent electrochemistry^48,49,66^ will be key.

Nonideal Arrhenius behavior, which would be apparent in temperature dependent activation energies and concave Arrhenius plots,^25,81^ is absent in our data. Instead, we observe linear fits with very high *R*^2^ linear regression values, in line with (quasi)equilibrium statistical mechanics. Therefore, in absence of deviations that require more elaborate kinetic models (with many more free tuning parameters), we currently refrain from invoking deviations from regular Boltzmann distributions^26^ or proton tunneling. Especially, the latter has been considered in electrocatalysis for decades, without any clear experimental evidence so far.^66^

## Conclusions

In conclusion, we are tempted to state that Tafel^82^ and Gileadi^83,84^ observed linear Tafel slopes for sp-metals over decades of current density, not because of simple Butler–Volmer type kinetics. Instead, sp-metals are governed by entropy-enthalpy compensation effects over the whole potential range.^21−24^ These important insights by Conway have been largely overlooked in recent decades which were dominated by the promotion of outer-sphere frameworks and oversimplified theory, with the adverse effect of isolating electrocatalyst research and education from other fields. In fact, to build an overarching bridge between electro-, thermal, and biocatalysis, partitioning of the activation free energy (Δ*G*) into Δ*S*(η) and Δ*H*(η) via potential and temperature dependent studies is critical. For example, in enzyme catalysis, recent studies highlight entropic changes during ion solvation, which can differ greatly between enzyme and water environments. The computational work by Aqvist and co-workers^85−87^ suggests that enzymes might be able to preorganize the solvent at the active site in three dimensions, leading to ion delocalization and, thus, an increasing activation entropy, however, without accruing an enthalpic penalty, enabling massive rate enhancements.

## Experimental Section

The methods are similar to the ones previously reported by us.^18^

### Membrane and Ionomers

Nafion 212 (Fuel Cell Store) (∼50 μm) was used as the cation-exchange layer (CEL). The as-received membranes were cut into 1.5 × 1.5 cm^2^ pieces and immersed for at least 24 h in deionized water before further use. PiperION-A25-HCO3 (∼25 μm thickness) and PiperION-A20-HCO3 (∼20 μm thickness) (Versogen) were used as anion exchange layers (AEL). The as-received membranes were cut into 1.5 × 1.5 cm^2^ pieces and immersed in 0.5 M KOH for at least 2 h after which the KOH was renewed, leaving the pieces in the solution for at least another 24 h. Nafion 520 (Sigma-Aldrich) was used as an ionomer to prepare the electrodes for the acidic experiments with the CEL and a dispersion of PiperION-A25-HCO3 in ethanol (Versogen) was used as the ionomer for the experiments in base using an AEL.

### Electrode Preparation

The gas diffusion electrodes (GDEs) were fabricated by spray-coating a dispersion containing the electrocatalyst and respective ionomers onto Freudenberg H23C2 (Fuel Cell Store). High-loading counter electrodes were prepared by mixing 100–150 mg of Pt/C 70% and 400–600 mg of the respective ionomer solution with 0.5 g of H_2_O and 1.7 g of isopropyl alcohol (IPA). The low-loading working electrodes were prepared by mixing 3–5 mg of different carbon-supported metal nanoparticles (see Supplementary Table 1 for reference information) with 12–20 mg of the respective ionomer solution and 0.5 g of H_2_O and 1.7 of IPA. The dry ionomer loading for both the working and counter electrodes was of 20% wt. The dispersions were sonicated for at least 4 h before spraying onto Freudenberg H23C2 cut to ∼5 × 5 cm^2^ and heated at 85 °C. The resulting total loading for the Pt/C counter electrodes ranged between 1.5–2 mg cm^–2^ while the working electrodes ranged between 10 and 90 μg cm^–2^. Finally, the GDEs were cut into 1 × 1 cm^2^ pieces to be used in the electrochemical setup. See Supplementary Tables 2 and 3 for further information about the working electrode loadings and measurement protocols. For all studies in the main manuscript, the PiperIon containing GDEs were ion-exchanged for 24 h ion KOH solution whereas the Nafion containing GDEs were used as prepared in their protonated form. Supplementary Figure 10 shows the HER kinetics for the PiperIon GDEs without additional hydroxide ion exchange (i.e., when the GDEs are assembles in the bicarbonate form). To test for anion adsorption, the PiperIon GDEs were soaked in 1 M H_2_SO_4_, HClO_4_ or KCl solution for 12 or 48 h as indicated.

### Membrane Electrode Assembly

The electrochemical measurements were performed in a regular membrane electrode assembly (MEA) in a fuel cell setup. First, a high loading PtC counter electrode is placed inside the gasketing space composed of two gaskets of 150 and 50 μm. Next, an ion-exchanged membrane (Nafion 212 for the experiments in acid and PiperION A25 or A20 (Versogen) in for the ones in alkali) is placed on top of the GDE, making sure that the electrode area is fully covered. The AELs were thoroughly washed and soaked in pure water before being placed on top of the electrode. Another two gaskets (150 and 50 μm) were placed on top before finally adding the working electrode facing the membrane. The whole stack was fixed with 8 screws with a torque at 4 NM. The MEA setup provides continuous physical compression between all layers during operation and enables a zero-gap contact between GDEs and ionically conductive membranes. As, allowing us to operate the stack in absence of liquid electrolytes, feeding only a pure-water humidified gas stream of H_2_.

### Electrochemical Setup and Initial Stabilization

The electrochemical experiments were performed using a Gamry Reference 3000 potentiostat with a fuel cell humidification system (Fuel Cell Technologies Inc., Model LFHS-C) that controlled the MEA cell temperature, as well as the humidification, flow rate, back-pressure and temperature of the H_2_ feed. Humidified H_2_ gas was fed to both, the counter and working electrodes (anode and cathode), operating the cell as H_2_ pump. Due to the fast kinetics of the HOR in the highly loaded (1.5–2 mg cm^–2^) PtC counter electrode, the cell potential informs directly on the HER kinetics of the low loading (10–100 μg cm^–2^) working electrodes.

For the acidic experiments using Nafion as CEL, humidified H_2_ gas heated to 65 °C was fed into the MEA cell through heated inlets at 70 °C (to prevent condensation) and at equal flow rates of 25 standard cubic centimeters per minute (SCCM) until a backpressure of 1 bar was obtained for both the counter and working electrode chambers. Then, the cell was heated from room temperature to 63.5 °C while applying a reductive potential in the working electrode to keep track of the current evolution during the heating process for a minimum of 40 min. The value of the reductive potential applied depended on the metal electrocatalyst at use to account for different stability windows.

For the experiments using PiperION as the AEL, the counter and working electrode chambers were connected to the same gas outlets as this showed to improve the stability of the measurements. To account for the water consumption in the working electrode during the HER reaction in base, the H_2_ fed to the working electrode was supplied at 75 °C through an inlet heated to 80 °C to increase the humidification of the gas. For the counter electrode, the humidified H_2_ was supplied at 65 °C. Gas was fed until a backpressure of 1 bar was obtained for the common chamber. A high working electrode flow of 80 SCCM was selected to provide a sufficient amount of water to the GDE where the water acts as a reactant in the HER reaction while keeping the flow of the counter electrode at 10 SCCM to avoid flooding since water is generated as a product of the HOR reaction in base. Finally, the cell was heated from room temperature until 63.5 °C while applying a reductive potential of −600 mV to keep track of the current evolution during the heating process for at least 60 min.

### Chronoamperometric and Impedance Temperature Measurements

Starting with the equilibrated cell at 63.5 °C after the initial heating and conditioning, an additional prestabilization chronoamperometry at the most reductive potential measured was applied for 15–60 min before collecting the data. Then, subsequent chronoamperometric and impedance measurements were performed, starting from the most reductive potential toward least reductive potentials (smaller overpotentials). Each chronoamperometric potential step was held between 1 and 5 min depending on the potential and current range, to ensure a stable steady-state performance. At the end of each chronoamperometric step, a potentiostatic electrochemical impedance spectroscopy (EIS) measurement with an amplitude of ±10 mV in the range of 1 Hz–10 MHz was done before changing the potential to the next chronoamperometric measurement. Once the chronoamperometries and EIS were collected, the temperature of the cell and the gases were lowered and the measurement protocol was repeated including the prestabilization step at the most reductive potential. The potentiostat was programmed to maintain the potential between chronoamperometries and EIS, avoiding the return of the system to open circuit. See Supplementary Table 3 for the potential steps.

The MEA nominal cell temperatures were 63.5, 53.5, 43.5, 33.5, and 25.0 °C. For the acidic Nafion experiments the H_2_ gas temperatures were 65, 55, 45, 35, and 25 °C for the counter and working electrode chambers and 70, 60, 50, 40, and 30 °C for the anticondensation gas inlets. For the basic PiperION experiments, the H_2_ working electrode gas bottle was kept 10 °C above the cell for the three hottest measurements, 75, 65, 55, 35, and 25 °C and the inlets at 80, 70, 60, 40, and 30 °C to avoid drying the membrane and the GDE in the electrode where H_2_O is consumed due to its participation in the HER in base. The temperature of the counter electrode inlet was identical to the values used in the acidic Nafion experiments. The temperature of the MEA cell was kept slightly below to maintain humidification in the GDEs and the membranes.

## Data Availability

The authors declare that the data supporting the findings of this study are available within the paper and its Supplementary files. Should any raw data files be needed in another format they are available from the corresponding author upon reasonable request.

## Supporting Information Available

Additional discussion, experimental details, and results (PDF)
