# Supplementary material for: Bias Dependence of the Transition State of the Hydrogen Evolution Reaction
Source: J Am Chem Soc. 2025 Feb 3;147(6):5472–85. doi: 10.1021/jacs.4c18638 (PMC11826909; doi:10.1021/jacs.4c18638)
Supplement: Supplementary file 1 — ja4c18638_si_001.pdf [file ja4c18638_si_001.pdf]

Supplementary Information

On the Bias Dependence of the Transition State of the Hydrogen Evolution Reaction

José M. Gisbert-González,[‡] Carlos G. Rodellar,[‡] Jody Druce, Eduardo Ortega, Beatriz Roldan Cuenya, Sebastian Z. Oener*

These authors contributed equally.[‡]

Department of Interface Science, Fritz-Haber Institute of the Max Planck Society, Berlin, 14195, Germany

Corresponding authors: oener@fhi-berlin.mpg.de

Supplementary Note 1

Capacitance measurements

The real part of the capacitance, $C_R(\omega) = -Z(\omega)_I \cdot (\omega \cdot |Z(\omega)|^2)^{-1}$, informs on the reversible amount of energy stored at the interface for a simple RC series circuit[1,2]. We refrain from fitting the data with traditional circuit models at this point. Our results strongly indicate that increasing excess charge at the surface can lead to increasing currents, due to a bias dependent activation entropy. This is not captured by regular parallel circuit models, where the Faradaic current is only dependent on the charge transfer resistance. On the other hand, extracting absolute capacitance values at lower frequencies is challenging without a more comprehensive circuit model. However, here we chose a high frequency of 1-10 kHz to compare variations of the real capacitance, $|\Delta C_R(\eta)|$, across the whole range of metals. We observe that $|\Delta C_R(\eta)|$ correlates closely with $\log_{10} \Delta A(\eta)$ and $\Delta E_A(\eta)$, with a R^2 value > 0.9 at 1-10 kHz. This is shown in Supplementary Figures 17-18 for the HER measurements with pristine and protonated Nafion GDEs and the pristine PiperIon GDEs, i.e. where the hydroxide ion exchange occurs during conditioning ($2H_2O + 2e^- \rightarrow H_2 + 2OH^-$). We also tested the impact of ion-exchanging the PiperIon GDEs prior to assembly *via* 12h exposure in 1M KOH. The different ion exchange conditioning leads only to smaller change in the bias dependent kinetics, as can be seen by comparing Figure 3 and Supplementary Figure 10. For the capacitance, we observe changes in the basic environment for Cu and Ni, possibly due to the reduction of a surface oxide layer during extended exposure to concentrated 1M KOH. Similarly, for Ni, we also observe capacitance changes, but less pronounced. Below, we first discuss the results of the capacitance when the ion exchange occurred during conditioning (after assembly) and then show summarized data for the case when the hydroxide ion exchange occurred before the assembly.

Supplementary Figure 19a shows the bias dependent $\log_{10} A(\eta)$ for Au and Cu with distinct slopes in acid and base. Consistent with the results in Fig. 2-3 (Supplementary Figure 10), the pre-exponential factor rises much faster in acid compared to base. Next, we extract the bias dependent capacitance $|\Delta C_R(\eta)|$ in acid and base. Due to the use of pure-water humidified membranes, specific ion adsorption is negligible, substantially simplifying the interpretation of the capacitance data. At electrochemical equilibrium the capacitance for the coinage metals is governed by the presence of water that leads to a small transfer of electronic charge from an adjacent water molecule to the electrode surface, which reduces the work function of the metal compared to vacuum and, thus, establishes the potential of zero charge[3,4]. Under bias, the capacitance is then primarily governed by water dipole reorientation[3] also observed *via* x-ray absorption[5] and vibrational sum frequency spectroscopy[6] and likely similar to sp-metals[7]. Additionally, there might be an impact of free H^+ and OH^- at the interface.

Supplementary Figure 19b shows that the capacitance varies substantially faster in acid compared to base. This different electric field response might be related to a fundamental difference[8] of hydroxide-water complexes in alkali[9,10], compared to proton-water complexes in acid[11–13]. Additionally, the proton concentration could increase and the distance to the electrode decrease, as suggested previously by Schmickler when discussing the impact of electrolyte anions[14]. This might additionally change the double layer capacitance, in contrast to base, where the water dipole might primarily respond by reorienting in the electric field. **Supplementary Figure 19c** shows the direct dependence of $\log_{10} A(\eta)$ on $|\Delta C_R(\eta)|$. Initially, $\log_{10} A(\eta)$ rises faster in acid than in base. However, at a bias of around ≥ 100 mV, the slope of $\log_{10} A(\eta)$ tends toward a constant value, which is substantially lower than the slope for $\log_{10} A(\eta)$ vs. $|\Delta C_R(\eta)|$ in alkaline electrolyte. However, the absolute values of $A(\eta)$ are

higher in acid than in base, implying a saturation behavior that is impacting an initially higher A(η) in acid at lower overpotentials than in base. Previously, saturating water dipole ordering at higher bias was predicted, e.g., *via* MD[15].

Supplementary Figure 19d-e shows the bias dependent pseudo-capacitance for the iron triad and PGMs, respectively. Consistent with our discussion and kinetic data in Fig. 2-4, in acid these metals reduce the activation enthalpy directly and bind hydrogen on their surface. As a result, besides the contribution from interfacial water dipole ordering, they also display larger pseudo-capacitive components that originate from the hydrogen coverage, in contrast to the coinage metals that are poor hydrogen binders in acid and alkali. In alkali, the iron triad shows the expected behavior as for the coinage metals, i.e. a much lower (pseudo-) capacitance is apparent due to the difficulty of extracting hydrogen from the electrolyte. As a result, the capacitance response is likely governed by water dipole reorientation, as for the coinage metals.

The exact absolute capacitance values depend on the exact (nanoparticle) catalyst structure and chemical composition. Despite this lack of resolution, our results clearly show that the *bias dependent* capacitive behavior of all these metals is similar in magnitude within and distinctly different between the metal groups in acid and alkali and mirrors the grouping in the *bias dependent* kinetic maps in Fig. 2-4 (Supplementary Figure 10). This finding is critical to understand the fundamental origin of the compensation slopes in acid and base. Higher pseudo-capacitive changes occur in acid for d-band metal catalysts that directly reduce the activation enthalpy and change the coverage of hydrogen intermediates on the surface, which leads to a (surface configurational) compensating decrease in the pre-exponential factor. In contrast, for all metals in alkali and the coinage metals in acid, the capacitance changes are lower and of similar magnitude. These observations conflict with pictures that try to reconcile slower HER kinetics only with a competing OH-coverage dependence and/ or surface configurational entropy changes that are specific to the surface chemistry and would likely lead to capacitance variations that are similar in magnitude to acid. Conversely, we assign the shared capacitive changes for the coinage metals and iron triad in alkali and the coinage metals in acid primarily to water dipole ordering, which impacts the solvation kinetics as discussed above. For these metals, **Supplementary Figure 19f** compares the slope $\Delta\log A(\eta)\cdot\Delta E_A(\eta)^{-1}$ in acid and base vs. the changes of the pre-exponential factor with capacitance, $\Delta\log A(\eta)\cdot|\Delta C_R(\eta)|^{-1}$. Due to the linear compensation between logA and E_A, the results in Supplementary Figure 19f are also applicable to $\Delta E_a(\eta)\cdot|\Delta C_R(\eta)|^{-1}$. For PGM nanoparticles in alkali (Supplementary Figure 19e), we observe similar capacitive changes as for the other metals in alkali. However, we do not observe extended compensation slopes at low bias, likely due to the fundamentally different kinetic behavior of these metals (Supplementary Figure 10a and Fig 3a).

Supplementary Figure 20 shows the same analysis as Supplementary Figure 19, but with the PiperIon GDEs that were ion-exchanged with hydroxide ions before assembly and the same Nafion GDEs as in Supplementary Figure 19. Overall, the behavior is very similar, with the striking difference of a larger capacitance for Ni and a much larger capacitance for Cu in base. We hypothesize that this might be related to dissolution or the reduction of an oxide layer that forms during extended exposure to 1M KOH. Irrespectively, the results are qualitatively in agreement with Figure 19 and together with the comparison of the kinetics in Figure 3 and Supplementary Figure 10, strongly indicate that trace amounts of bicarbonate do not drastically impact the kinetics.

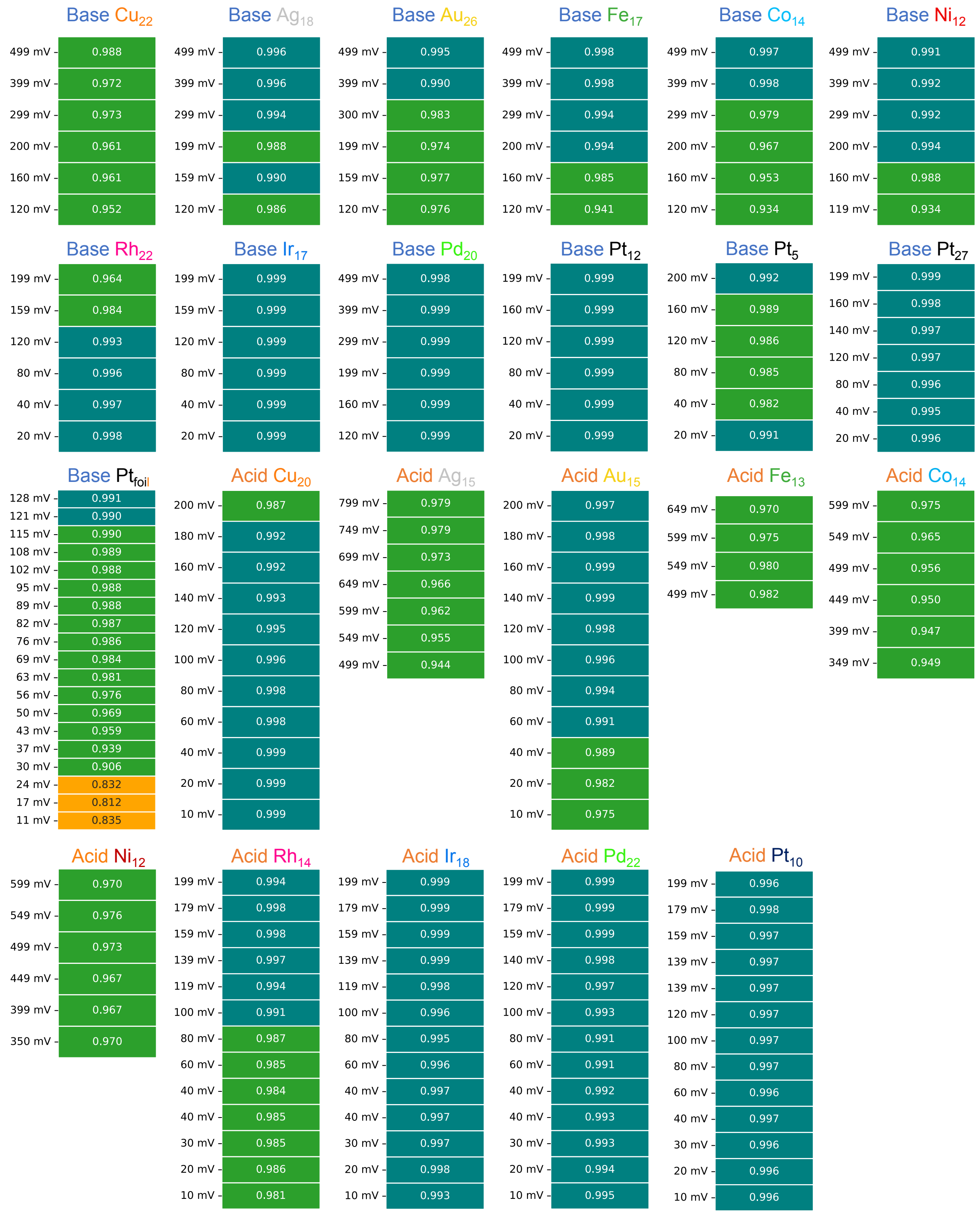

Supplementary Figure 1 | Heatmaps for R2 values for the Arrhenius linear regressions. The goodness of a linear regressions is generally estimated by the R^2 coefficient, which is a measure of how good the model explains the variance of the dependent variable (log10(j)) with respect to the independent variable (1/T). The precision of the linear regression, that is, the deviation of the points from the trendline, is estimated with the standard error of the regression (S), which is plotted in each kinetic map as the error bars of $\log_{10}(A)$ and E_A. Except for the Pt foil, the R^2 coefficients of the MEA experiments are above 0.98, showing the advantages of using this setup to perform temperature-dependent studies. For the majority of experiments, five temperatures were used for the Arrhenius fit. The color code is used to emphasize the different R^2 values.

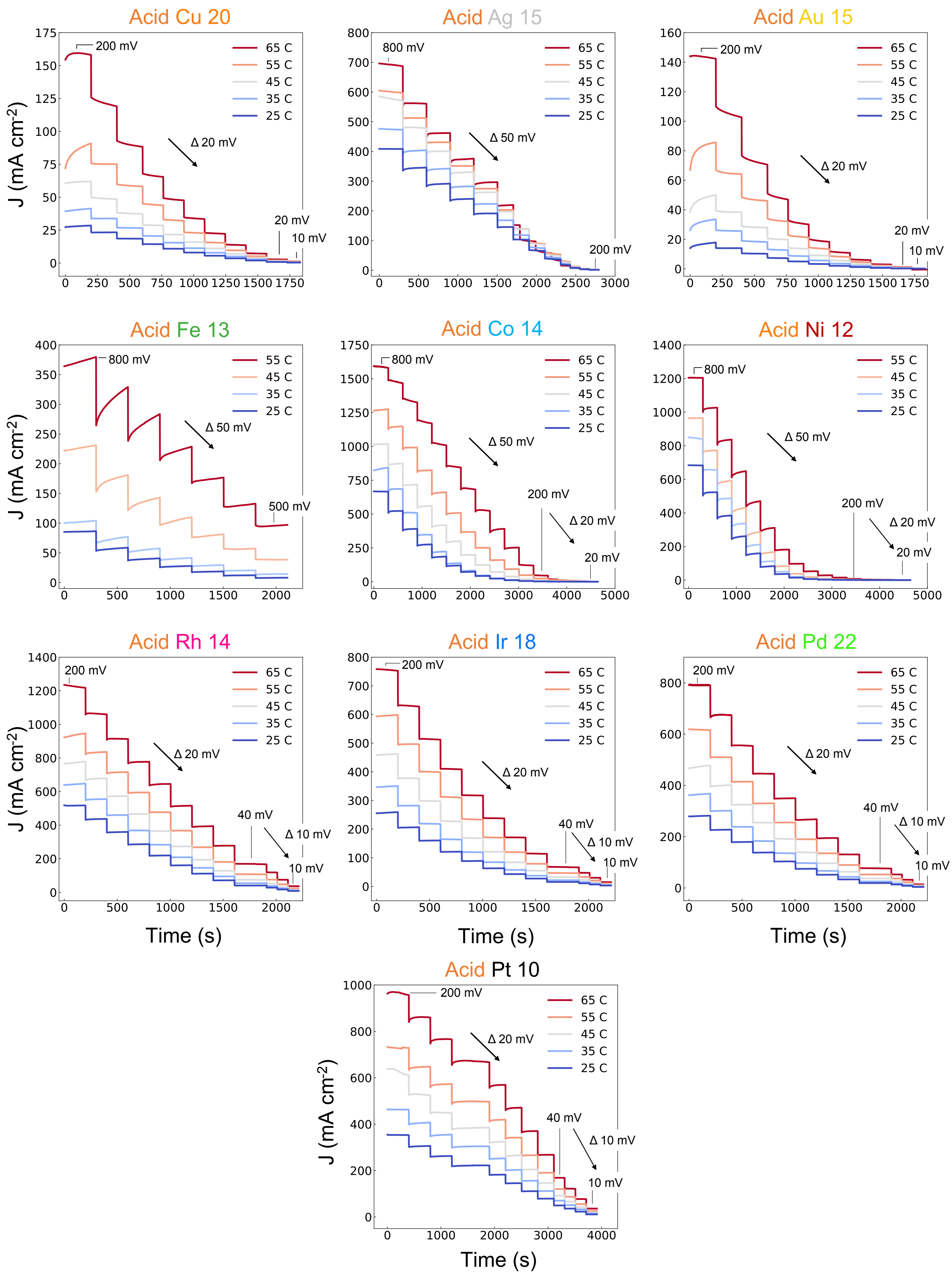

Supplementary Figure 2 | Chronoamperometric HER studies on acidic Nafion. Before performing the multi-step chronoamperometry shown here, the potential was held for 15-60 min at the value of the first potential step to allow for conditioning. Between the potential steps shown, potentiostatic electrochemical impedance was measured in the range of 1-10^6 Hz. The nanoparticle loading is shown next to each metal in μg cm^{-2}. See Supplementary Table 2 for details on the dispersion.

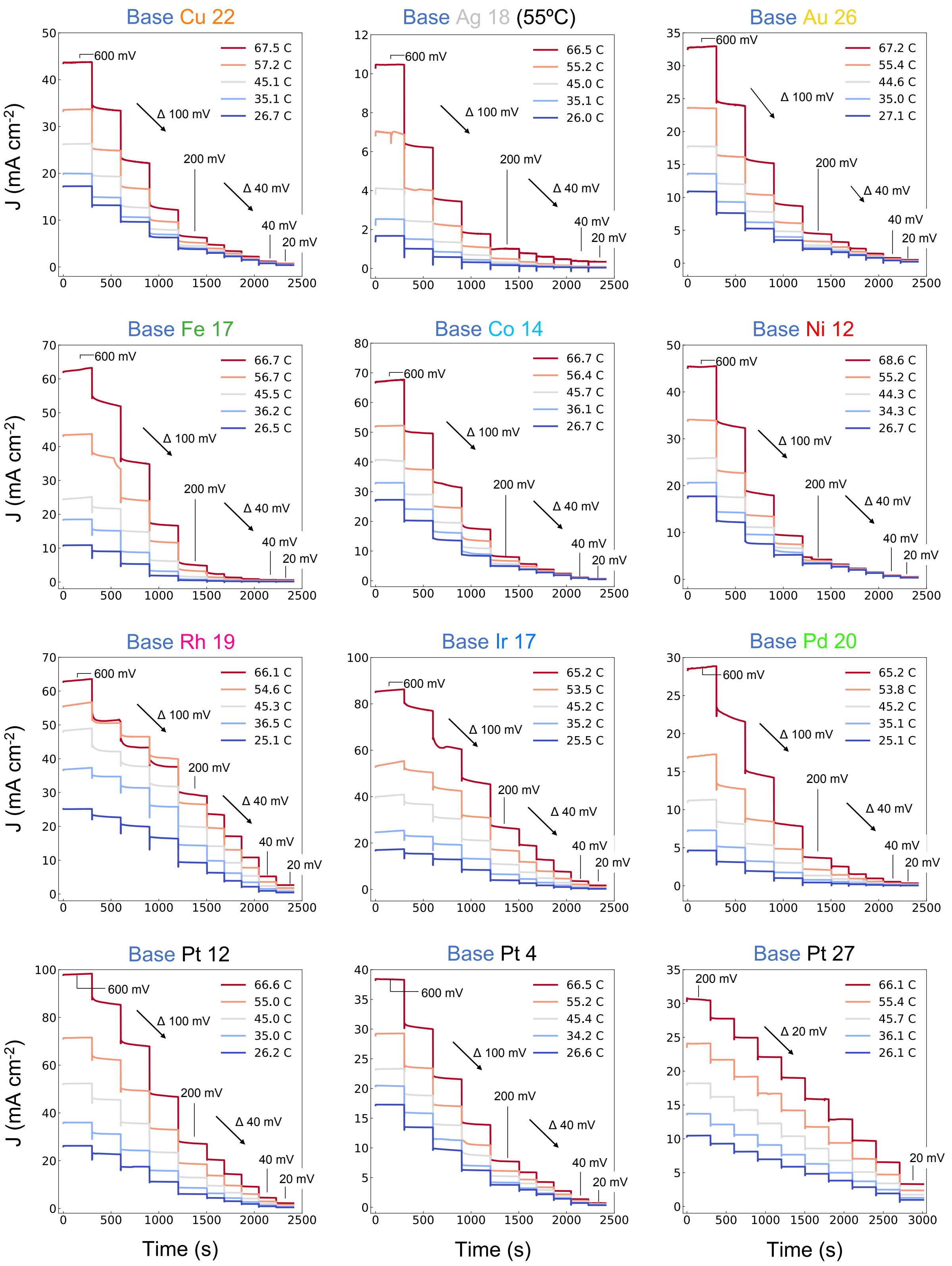

Supplementary Figure 3 | Chronoamperometric HER studies on alkaline PiperION A. Before performing the multi-step chronoamperometry shown here, the potential was held for 15-60 min at the value of the first potential step to allow for conditioning. Between the potential steps shown, potentiostatic electrochemical impedance was measured in the range of 1-10^6 Hz. The nanoparticle loading is shown next to each metal in µg cm^{-2}. See Supplementary Table 2 for details on the dispersion.

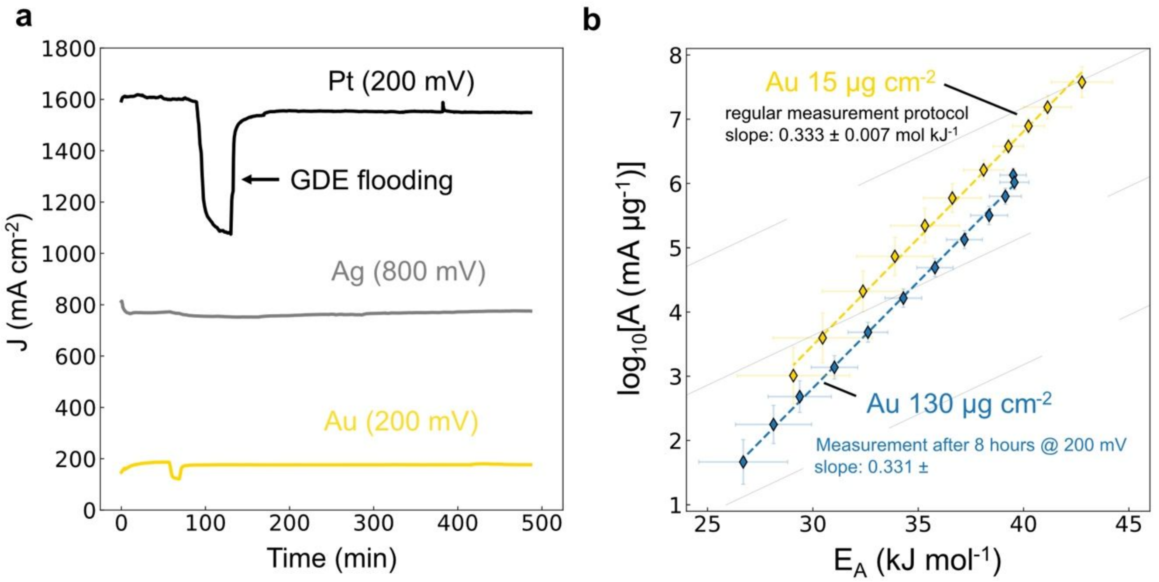

Supplementary Figure 4 | Durability test of hydrogen evolution reaction in membrane electrode assembly. a, Eight-hour durability test of three selected nanoparticles on Nafion at 65˚C, showing stable performance. For Pt/c we encountered a sudden dip in the current before full recovery which we assign to water transport issues in the hydrogen pump cell at high current densities. The solvated protons drag a lot of water toward the HER cathode, which can lead to flooding, especially at the high current densities for Pt/c. **b,** Exemplary pre-exponential factor (log A) and activation energy (E_A) for Au/c extracted from temperature dependent multistep chronoamperometry (see Methods for details) before and after the 8h durability test, confirming the robust measurements, especially with regard to the compensation slopes.

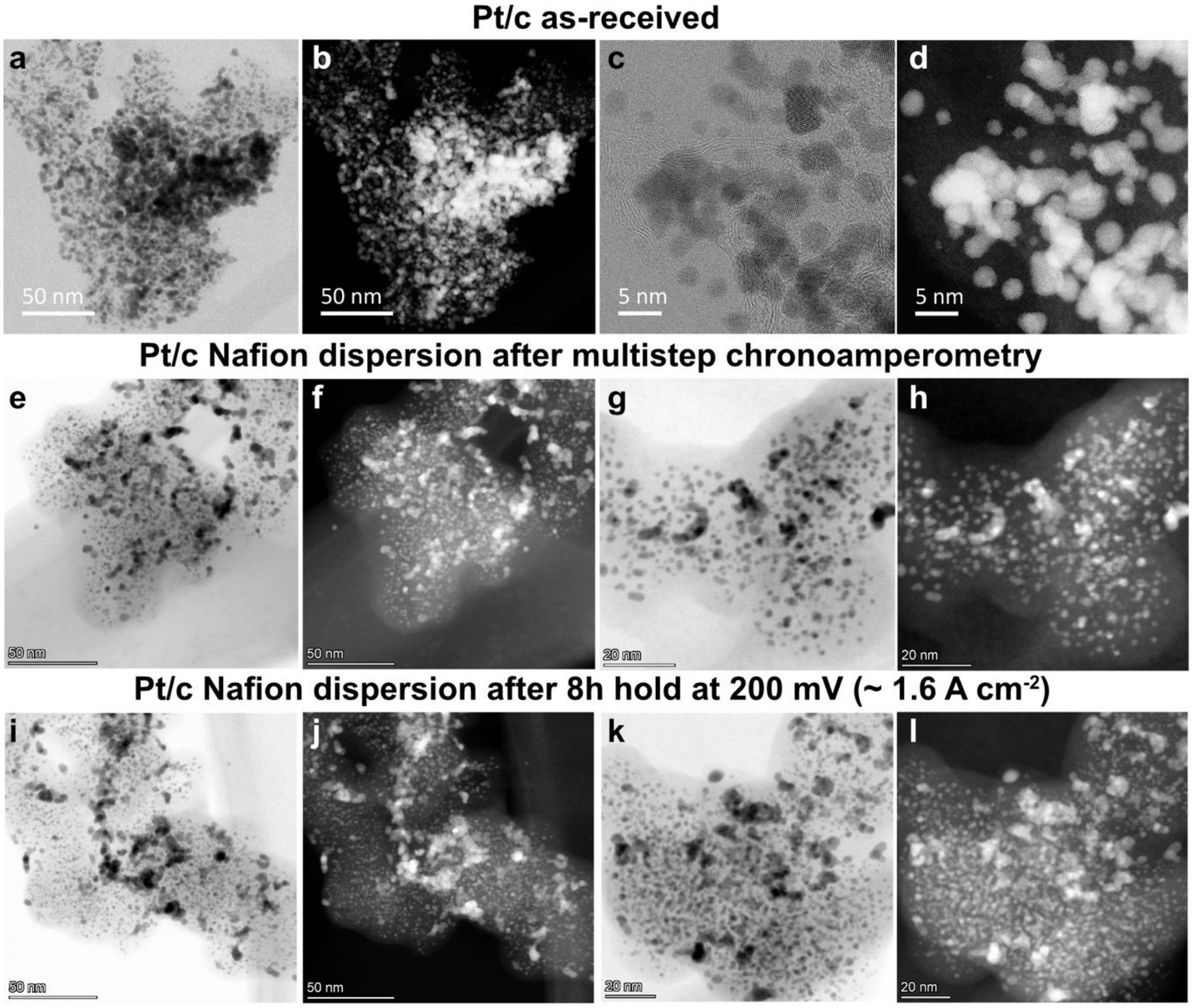

Supplementary Figure 5 | Before and after transmission electron microscopy of Pt/c. a-d, bright and dark field images of as-received Pt/c (without Nafion dispersion). **e-h,** bright and dark field images of Pt/c after multistep chronoamperometry on acidic Nafion (Supplementary Figure 2). **i-l,** bright and dark field images of Pt/c after 8h hold at 200 mV (~ 1.6 A cm^{-2}) on acidic Nafion (Supplementary Figure 4). For the electron microscopy after the electrochemical studies, the gas diffusion electrodes were sonicated in organic solvent to detach the nanoparticles before washing in alcohol. Unavoidably, some Nafion dispersion remained on the Pt/C nanoparticles, preventing high resolution images as in c-d. Irrespectively, the Pt/c nanoparticles remained well-dispersed in the carbon network even after the 8h test, in line with the stable performance in Supplementary Figure 4.

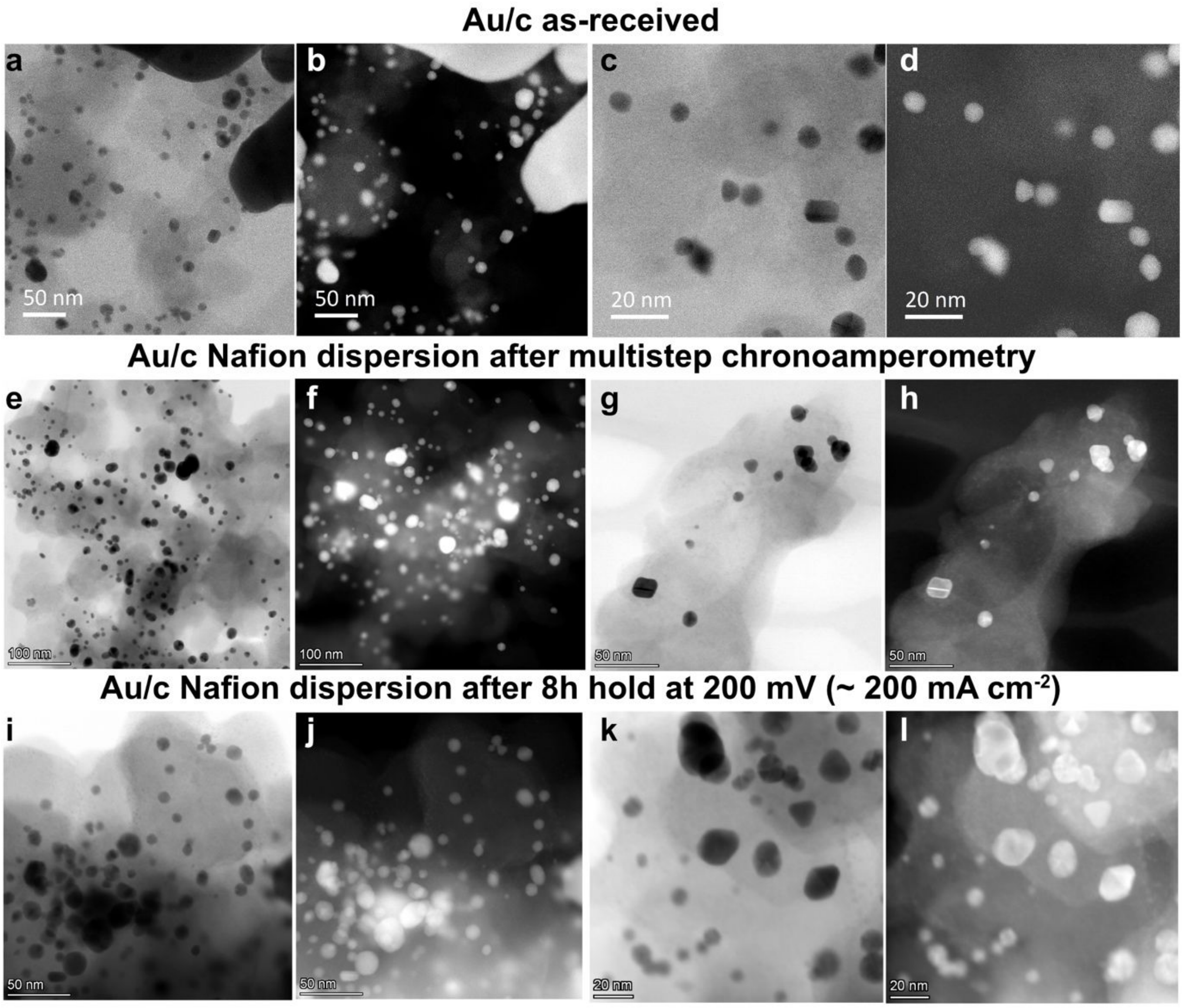

Supplementary Figure 6 | Before and after transmission electron microscopy of Au/c. a-d, bright and dark field images of as-received Au/c (without Nafion dispersion). **e-h,** bright and dark field images of Au/c after multistep chronoamperometry on acidic Nafion (Supplementary Figure 2). **i-l,** bright and dark field images of Au/c after 8h hold at 200 mV (~ 200 mA cm^{-2}) on acidic Nafion (Supplementary Figure 4). For the electron microscopy after the electrochemical studies, the gas diffusion electrodes were sonicated in organic solvent to detach the nanoparticles before washing in alcohol. Unavoidably, some Nafion dispersion remained on the Au/c nanoparticles, preventing high resolution images as in c-d. Irrespectively, the Au/c nanoparticles remained well-dispersed and did not substantially agglomerate or dissolve in the carbon network even after the 8h test, consistent with the stable performance in Supplementary Figure 4.

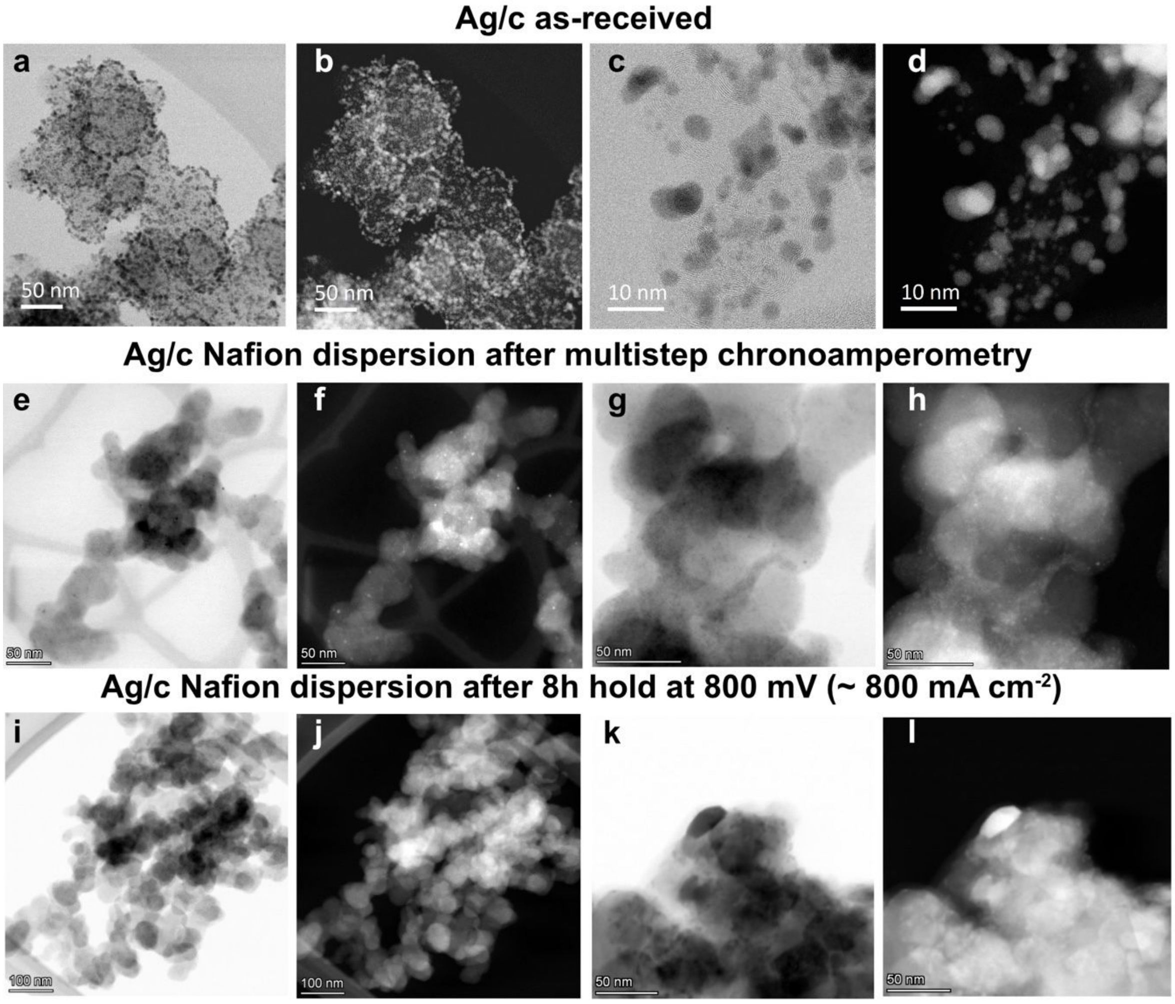

Supplementary Figure 7 | Before and after transmission electron microscopy of Ag/c. a-d, bright and dark field images of as-received Ag/c (without Nafion dispersion). **e-h,** bright and dark field images of Ag/c after multistep chronoamperometry on acidic Nafion (Supplementary Figure 2). **i-l,** bright and dark field images of Au/c after 8h hold at 800 mV (~ 800 mA cm^{-2}) on acidic Nafion (Supplementary Figure 4). For the electron microscopy after the electrochemical studies, the gas diffusion electrodes were sonicated in organic solvent to detach the nanoparticles before washing in alcohol. Unavoidably, some Nafion dispersion remained on the Ag/C nanoparticles, preventing high resolution images as in c-d. Irrespectively, the Ag/c nanoparticles show structural changes after the measurements. In e-h and k-l, we observe distinct small Ag nanoparticle clusters inside the Nafion/carbon network. However, in panel i-j, we observe primarily larger agglomerates. These changes might be linked to the instabilities during multistep chronoamperometry measurements at lower potentials (Supplementary Figure 2). Therefore, we limited our analysis of the temperature dependent rates to higher potentials, for which we observed stable performance. Most importantly, despite these apparent structural changes, the HER currents are clearly stable for 8h (Supplementary Figure 4), enabling reliable Arrhenius analysis.

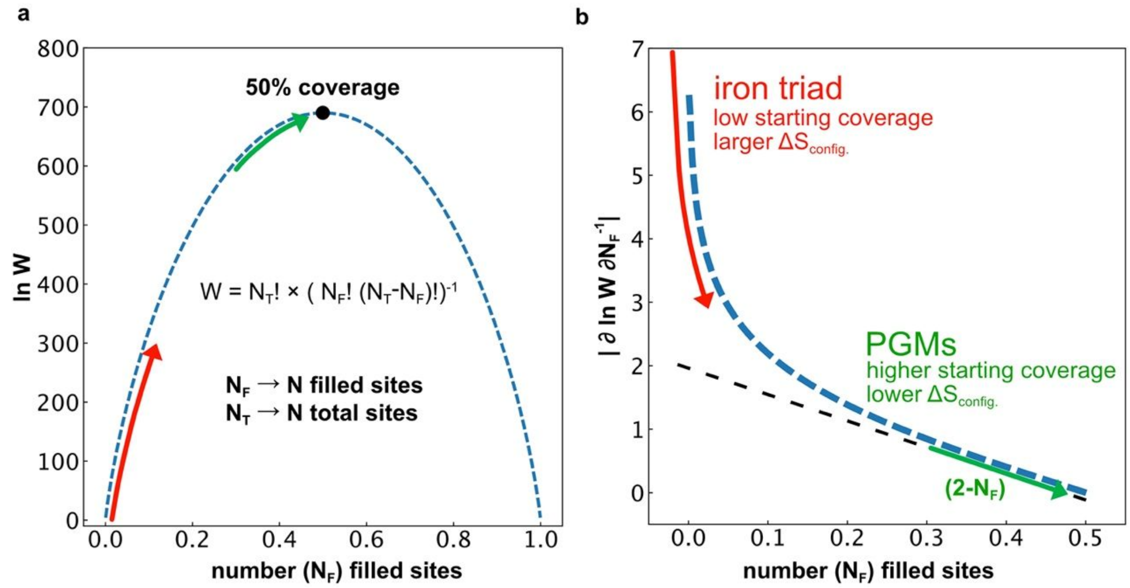

Supplementary Figure 8 | Surface configurational entropy changes for PGMs and iron triad. The configurational phase space is dependent on the bias dependent hydrogen coverage on the surface. **a,** The number of microstates (W) is highest for 50% coverage of available sites. **b,** log A is proportional to the incremental change of the number of microstates with increasing change of filled sites (slope in panel a). For the iron triad, these changes exceed the ones of the PGM. The PGMs contain a larger concentration of underpotential deposited hydrogen that participates in the reaction which might lead to larger effective concentration at equilibrium and, thus, lower changes in log A, as indicated in panel b. In contrast the iron triad is dominated by the (too) strongly bound underpotential deposited hydrogen at equilibrium. Conversely, the reaction might initially proceed solely *via* overpotential deposited hydrogen on the nanoparticles, leading to a lower effective hydrogen concentration at equilibrium and larger log A changes. Additionally, when stepping down from high negative overpotentials to less negative overpotentials, the hydrogen coverage on the iron triad might be increasingly impacted by parallel catalyst dissolution. For the PGMs, the changes of log A can be approximated with $\partial lnW \cdot \partial N_F^{-1} \propto (2 - N_F)$ for the chosen coverage range or even less when approaching a 50% covered surface.

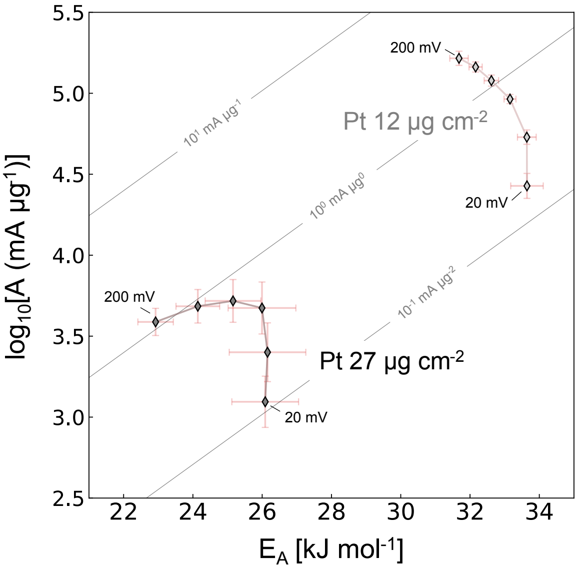

Supplementary Figure 9 | Kinetic map for different loadings of PtC in base. The exact metal loading can be seen as a subscript next to the Pt symbol in units of µg cm^{-2}. While the value of $\log_{10}$A and E_A is loading-dependent, the Pt 12 µg cm^{-2} measurements for Pt_{12} were selected for the main discussion due to the similar loading to the electrodes in acid of 10 µg cm^{-2}.

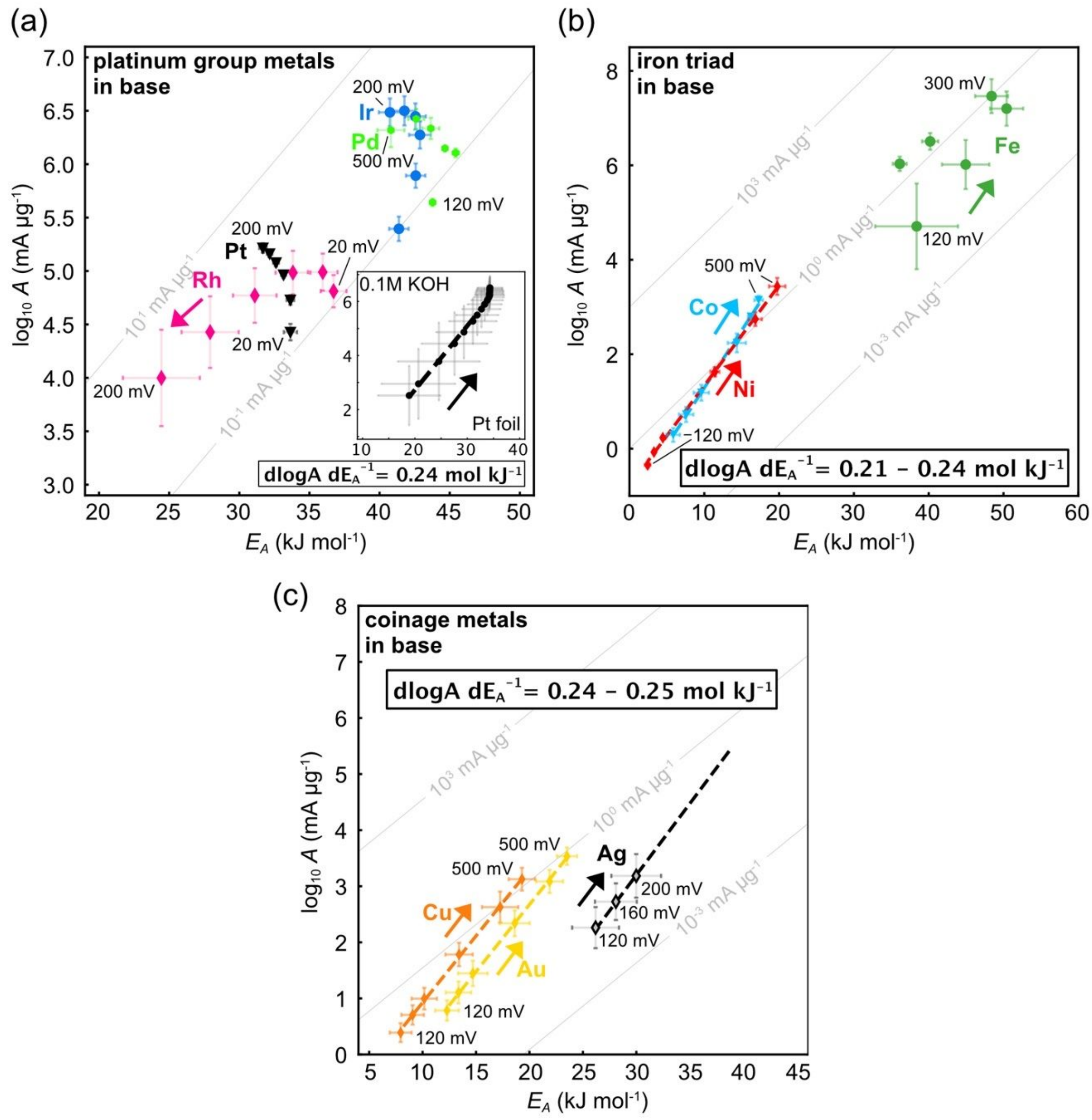

Supplementary Figure 10 | Bias dependent pre-exponential factor and activation energy for the hydrogen evolution reaction in alkali (without additional hydroxide-exchange before assembly). **(a, b)** logA vs. E_A for PGMs and iron triad in alkali, respectively. Reference measurements on polycrystalline Pt foils in 0.1M KOH (inset in (a)) result in the same compensation slope as for the other metals in base. **(c)** logA vs. E_A for coinage metals in alkali with a very similar compensation slope as the d-band metals in alkali (panel (b)), but substantially lower compared to the slopes of the coinage metals in acid of $\Delta \log A(\eta) \cdot \Delta E_A(\eta)^{-1} \sim 0.37 - 0.41 \text{ mol kJ}^{-1}$ (Fig. 2c). All listed potentials are cathodic overpotentials. For all catalysts, a low loading of 5-50 μg_{metal} cm^{-2} was used to limit the impact of mass transport and interfering kinetics of the internal reference electrode. Values are means and error bars reflect standard deviation for E_A (slope) and A (intercept) from Arrhenius analysis, based on five observations (temperatures). The grey diagonal lines in the background are iso-current lines at 65 ˚C, i.e. pairs of log A and E_A that result in the same current. For a-c, the GDEs were not ion exchanged with hydroxide ions prior to assembly, but remaining bicarbonate ions in the GDE are displaced with hydroxide ions generated during the conditioning after assembly ($H_2O + 2e^- \rightarrow H_2 + 2OH^-$). The kinetics are very similar to the ones in Fig. 3 of the main manuscript, further supporting the statistical robustness provided by the high R^2 linear regression values of the MEA to study HER kinetics.

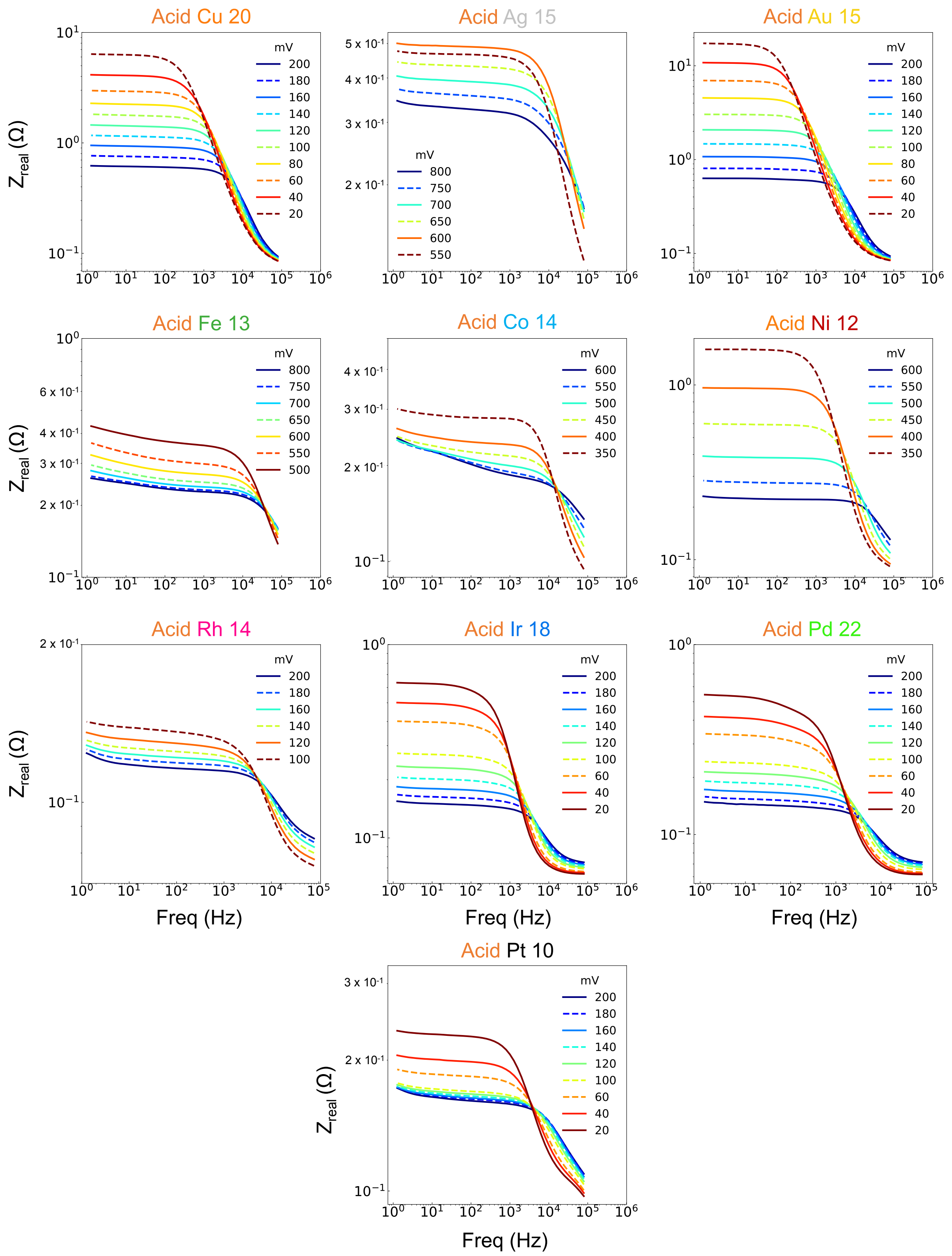

Supplementary Figure 11 | Real impedance Bode plots for the experiments in acid with Nafion. The potential dependent impedance measurements were performed at the end of each individual potential hold during the multistep chronoamperometry. For more details see Methods section.

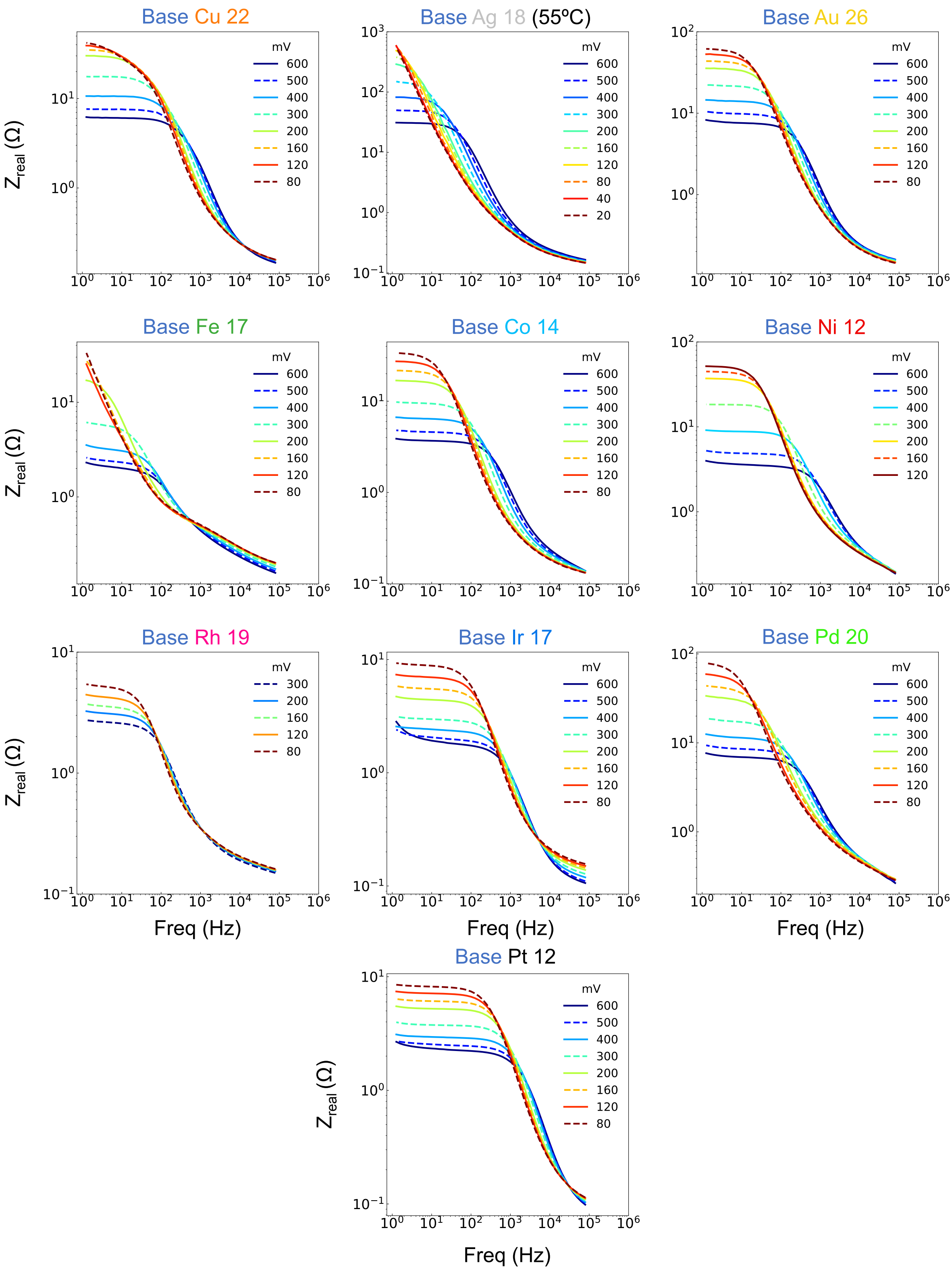

Supplementary Figure 12 | Real impedance Bode plots for the experiments in base with PiperION. The potential dependent impedance measurements were performed at the end of each individual potential hold during the multistep chronoamperometry. For more details see Methods section.

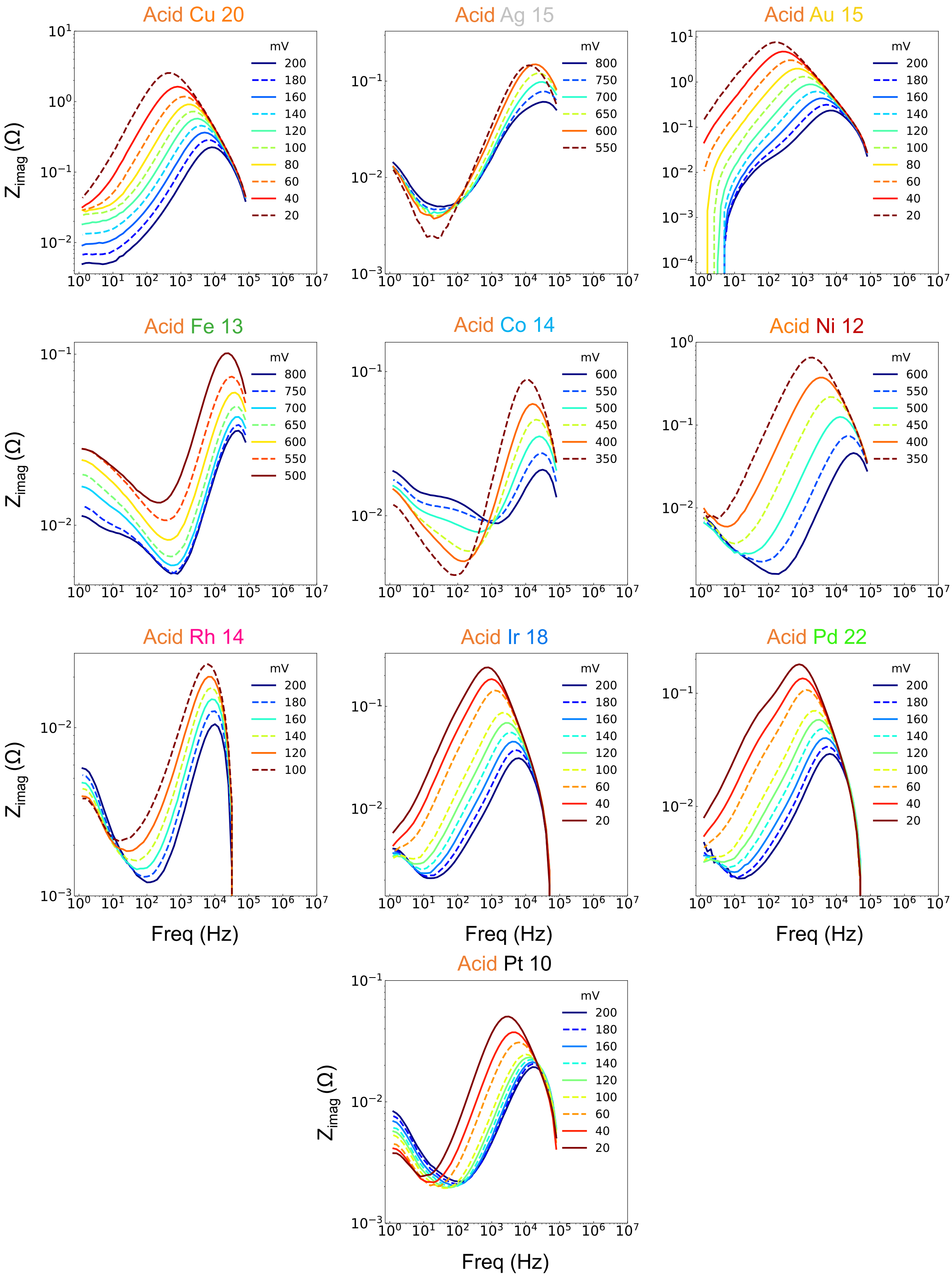

Supplementary Figure 13 | Imaginary impedance Bode plots for the experiments with Nafion. The potential dependent impedance measurements were performed at the end of each individual potential hold during the multistep chronoamperometry. For more details see Methods section.

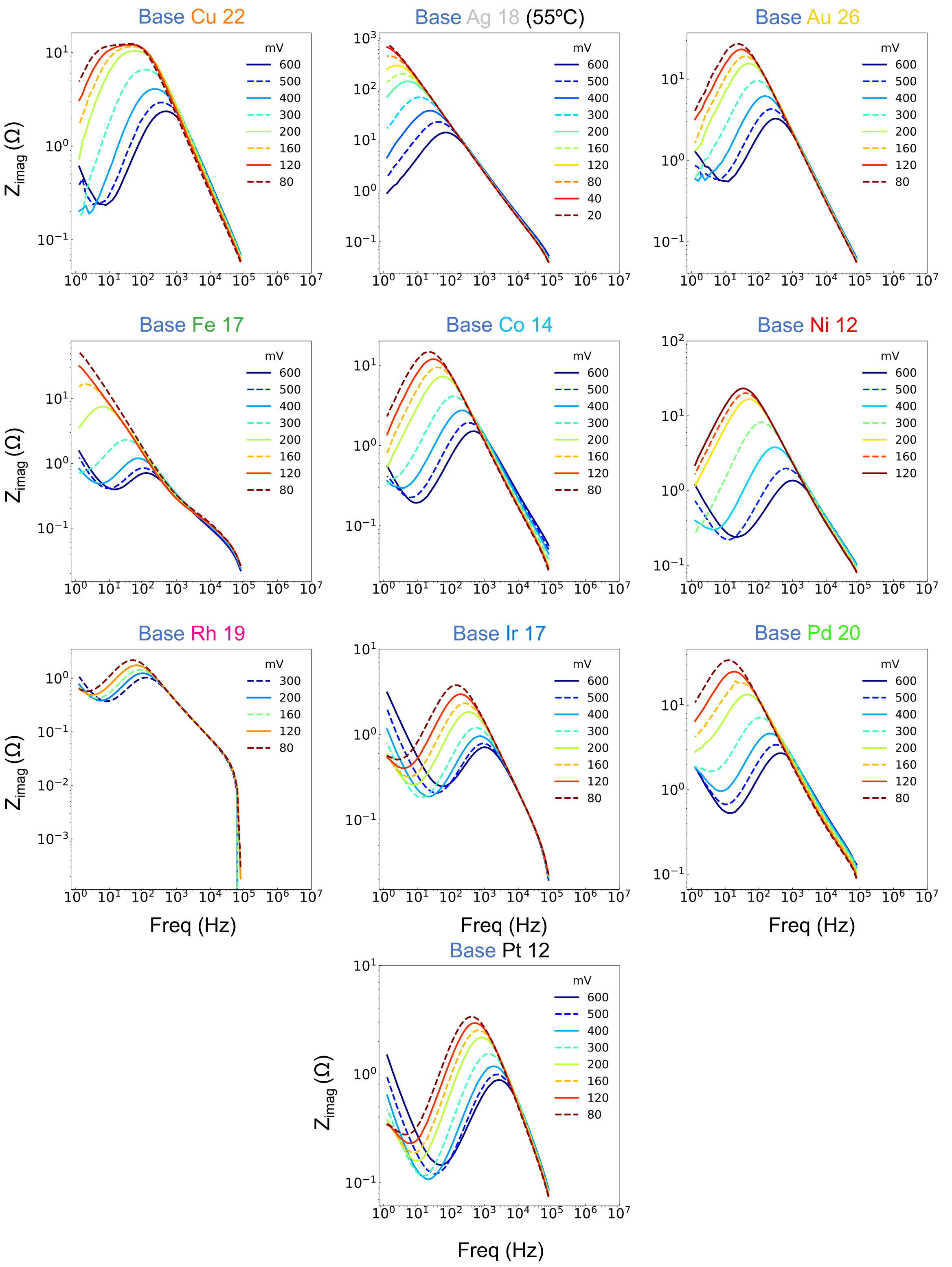

Supplementary Figure 14 | Imaginary impedance Bode plots for the experiments in base with PiperION. The potential dependent impedance measurements were performed at the end of each individual potential hold during the multistep chronoamperometry. For more details see Methods section.

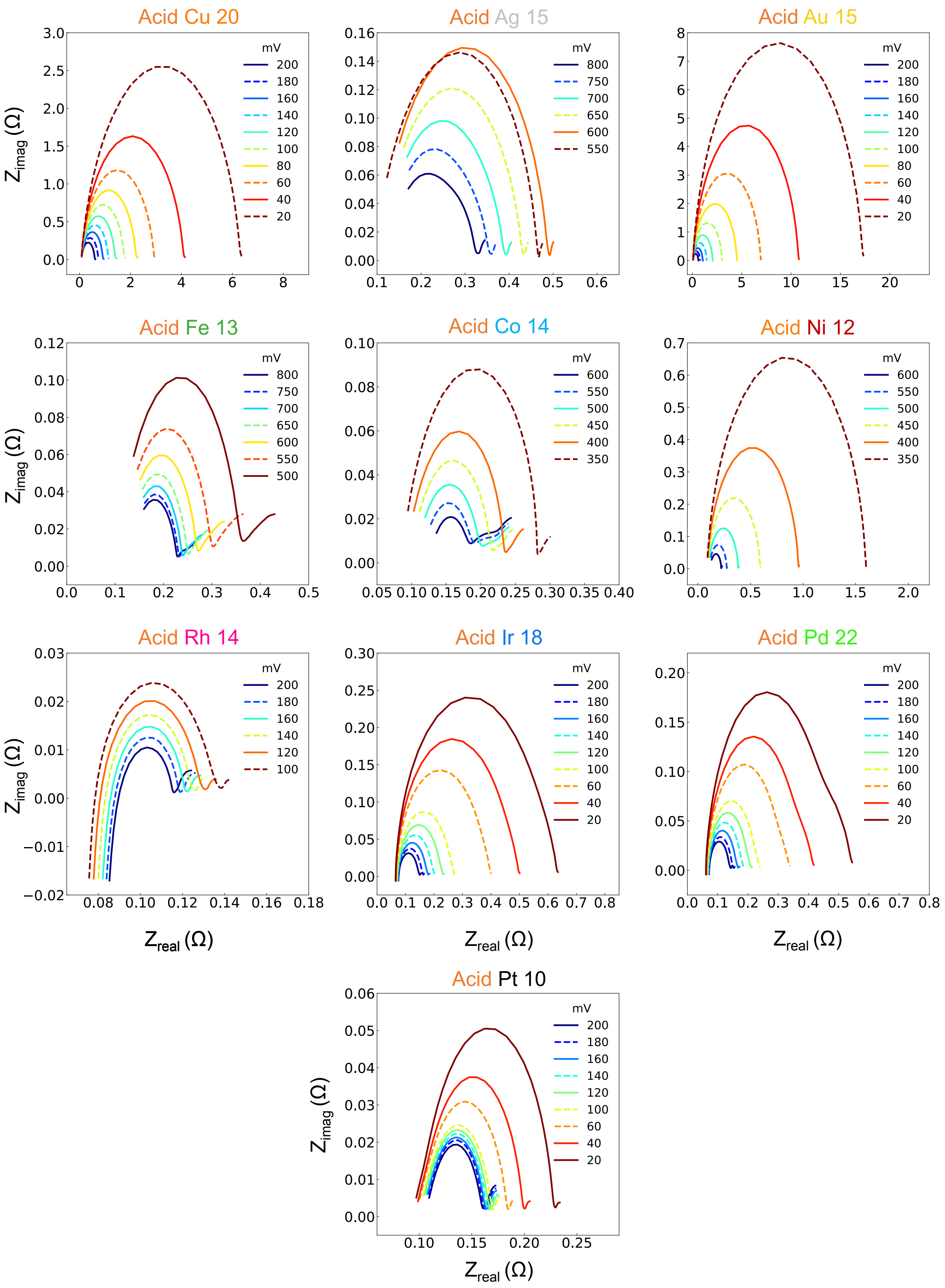

Supplementary Figure 15 | Impedance Nyquist plots for the acid experiments with Nafion. The potential dependent impedance measurements were performed at the end of each individual potential hold during the multistep chronoamperometry. For more details see Methods section.

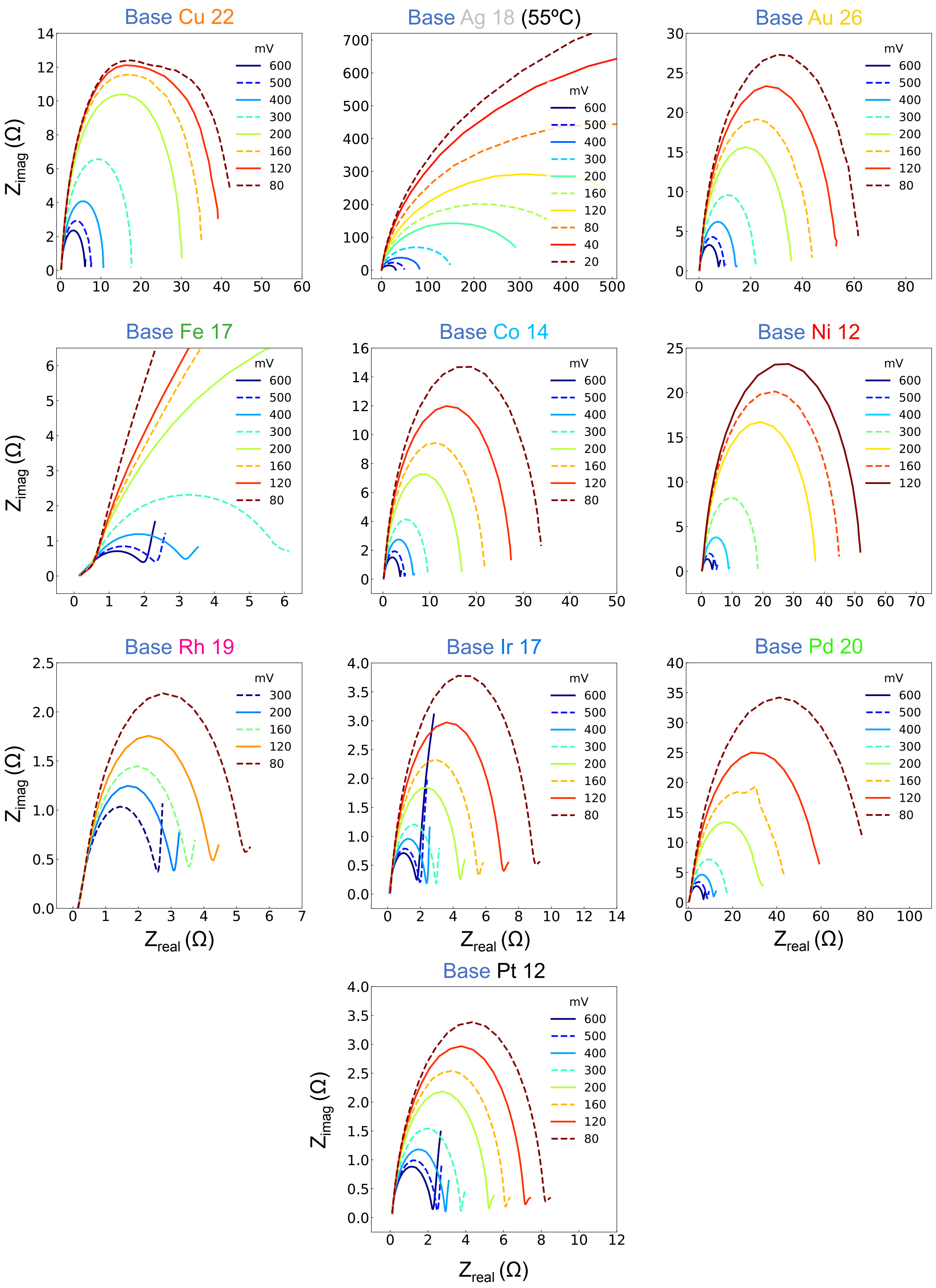

Supplementary Figure 16 | Impedance Nyquist plots for the base experiments with PiperION between 1-10^5 Hz. The potential dependent impedance measurements were performed at the end of each individual potential hold during the multistep chronoamperometry. For more details see Methods section.

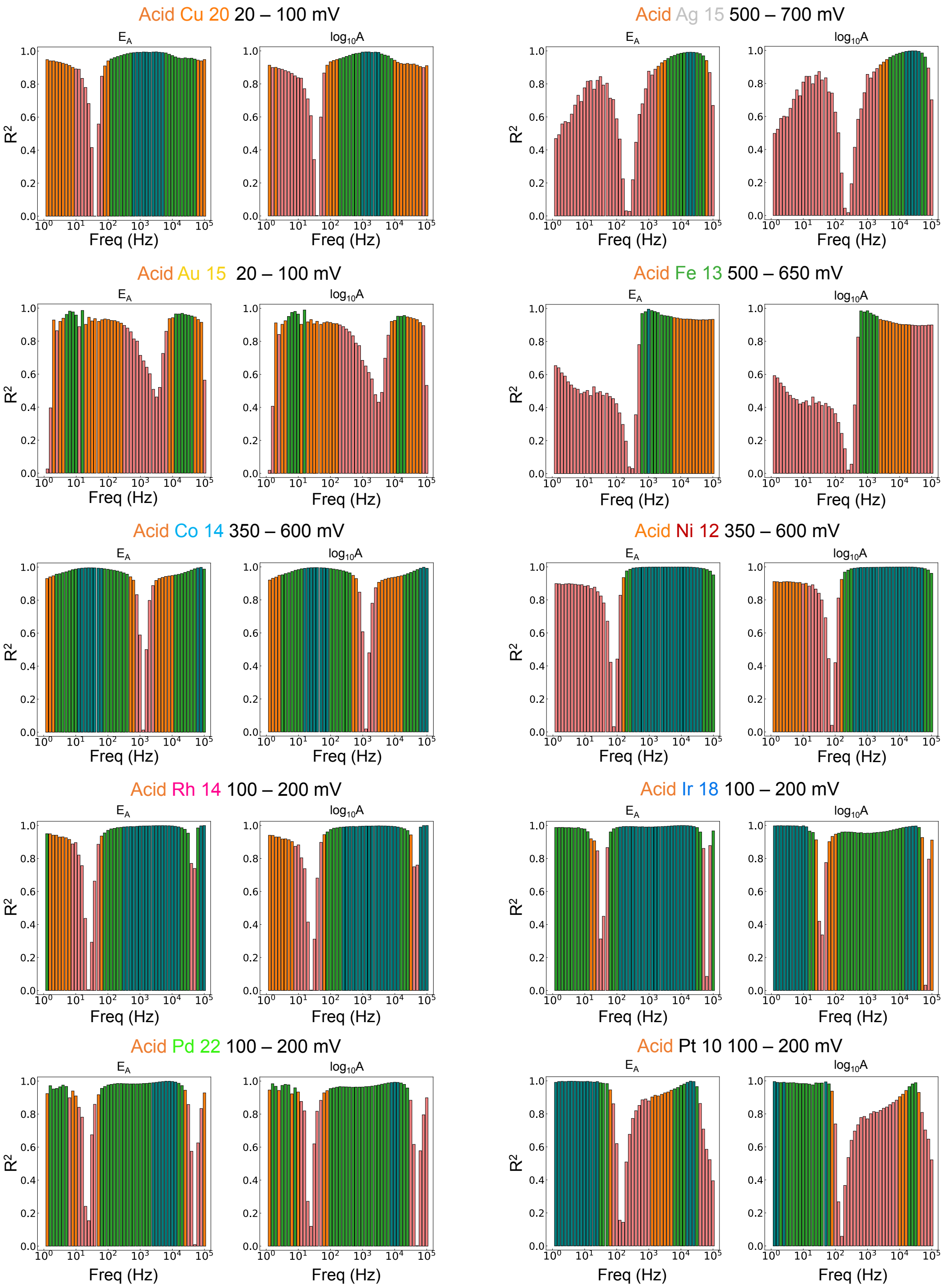

Supplementary Figure 17 | Correlation of real capacitance (C_R) at 65°C with Arrhenius parameters ($log_{10}A$, E_A) for different electrocatalytic interfaces in acid (Nafion). For the potential range where compensation effects are observed, the Arrhenius parameters and C_R correlate strongly at high frequencies for the different electrocatalysts. This allows us to compare these values at a chosen frequency of 10 KHz, where the response is mainly determined by the working electrode interface without fitting a circuit model where the faradaic current only depends on the charge transfer resistance and is independent of the capacitance. The color code helps to highlight the difference between R^2 values ($R^2 > 0.99$ dark-green, $R^2 > 0.95$ green, $R^2 > 0.9$ orange, $R^2 < 0.9$ red). The metal loading (without ionomer and carbon), is indicated for each experiment as a subscript next to the metal in µg cm^{-2}. The number of points used for each fit depends on the potential range used for each metal.

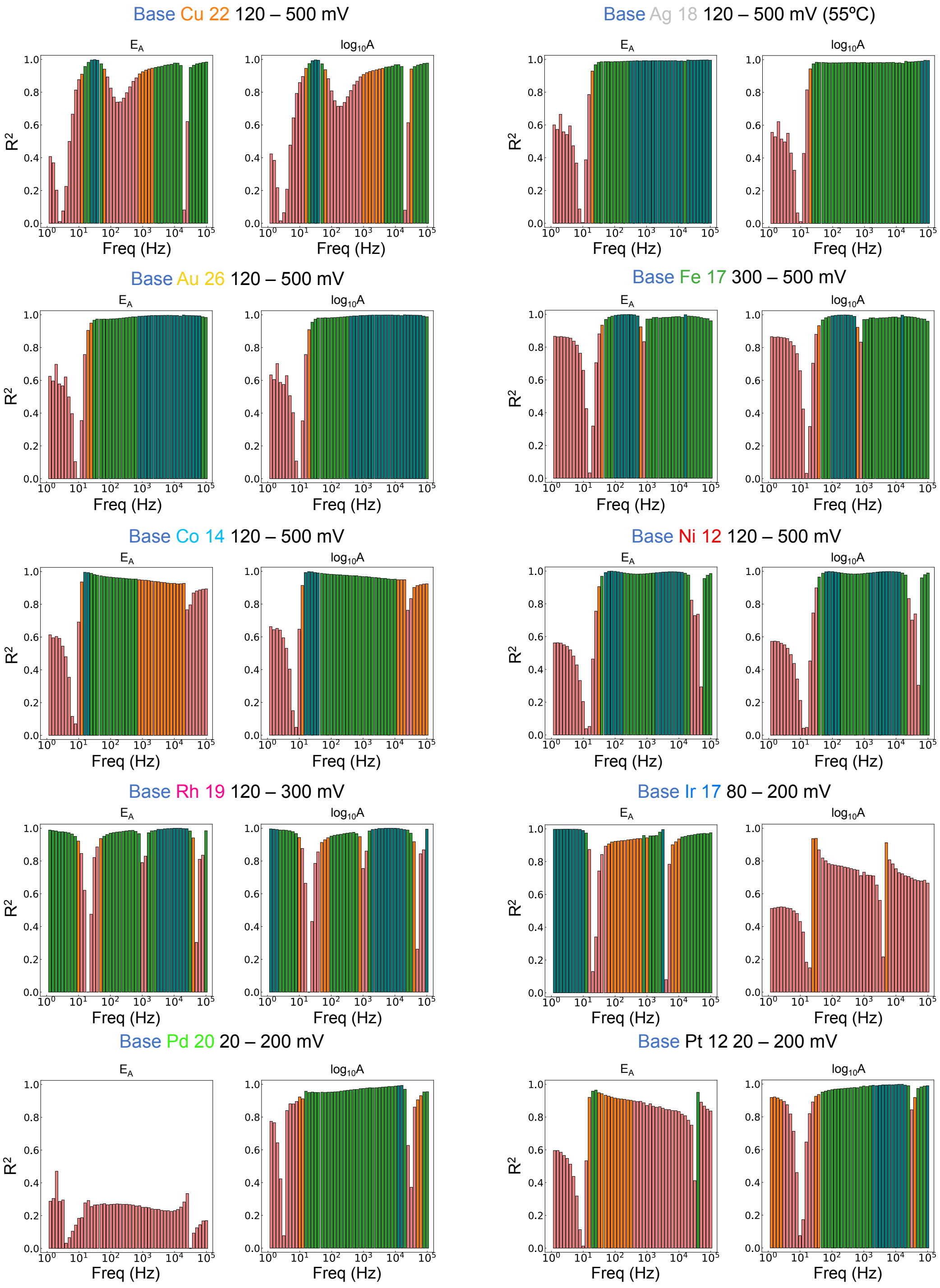

Supplementary Figure 18 | Correlation of real capacitance (C_R) at 65ºC with Arrhenius parameters ($log_{10}A$, E_A) for different electrocatalytic interfaces in base (PiperION). For the potential range where compensation effects are observed, the Arrhenius parameters (except for Fe, Ir, Pd, and Pt), and C_R correlate strongly at high frequencies throughout different electrocatalytic interfaces. This allows to compare these values at a chosen frequency of 10 kHz where the response is mainly determined by the working electrode interface without fitting a circuit model where the faradaic current only depends on the charge transfer resistance and independent of the capacitance. The color code highlights the difference between R^2 values (R^2 > 0.99 dark-green, R^2 > 0.95 green, R^2 > 0.9 orange, R^2 < 0.9 red). The metal loading (without ionomer and carbon), is indicated for each experiment as a subscript next to the metal in μg cm^{-2}. The number of points used for each fit depends on the potential range used for each metal.

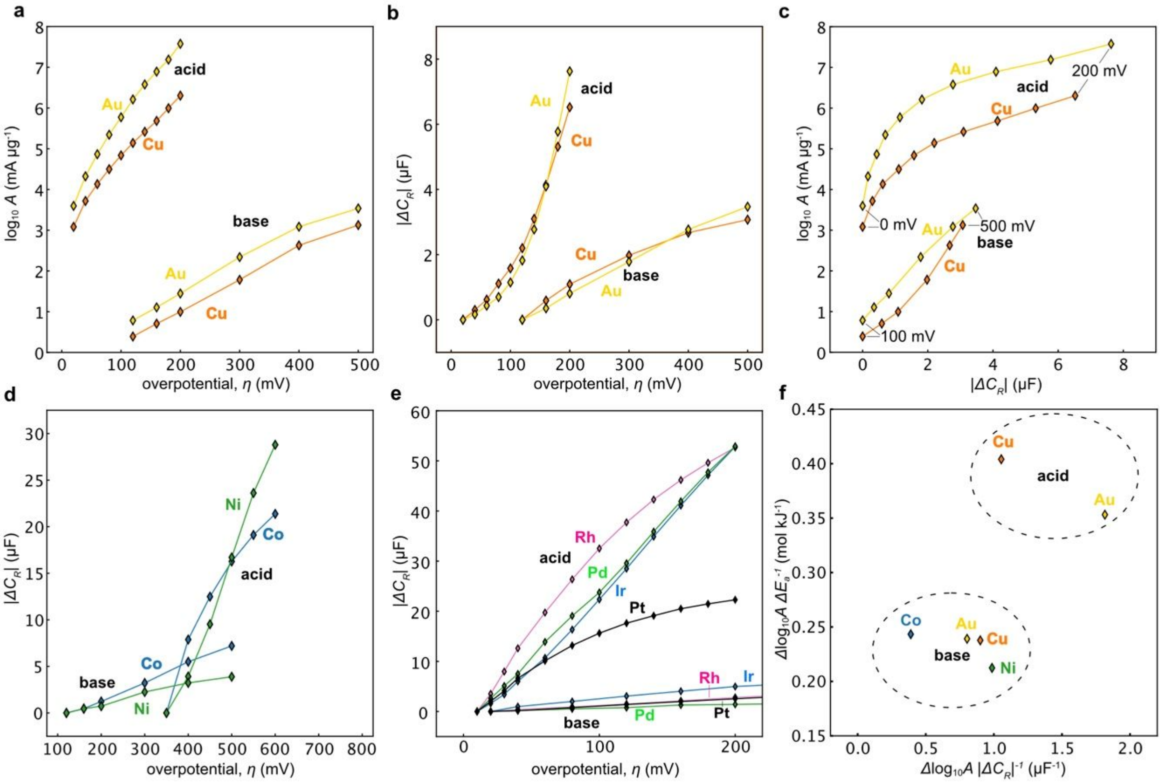

Supplementary Figure 19 |. The role of potential dependent capacitance in the hydrogen evolution kinetics. For the data in base, the GDEs were ion exchanged with hydroxide ions during the conditioning and after assembly ($H_2O + 2e^- \rightarrow H_2 + 2OH^-$). See Supplementary Figure 20 for hydroxide exchange before the HER. (**a-c)** Bias dependent pre-exponential factor, $\log A(\eta)$, absolute changes of the real capacitance, $|\Delta C_R(\eta)|$, and dependence of pre-exponential factor on absolute capacitance changes for Au and Cu in acid and base, respectively. **(d-e)** $|C_R(\eta)|$ for Ni and Co in acid and base and PGMs in acid and base. In acid the values are higher than for coinage metals, likely due to pseudo-capacitance related to hydrogen coverage. (**f)** $\Delta\log A(\eta)\cdot\Delta E_A(\eta)^{-1}$ vs. $\Delta\log A(\eta)\cdot|\Delta C_R(\eta)|^{-1}$ for selected catalysts.

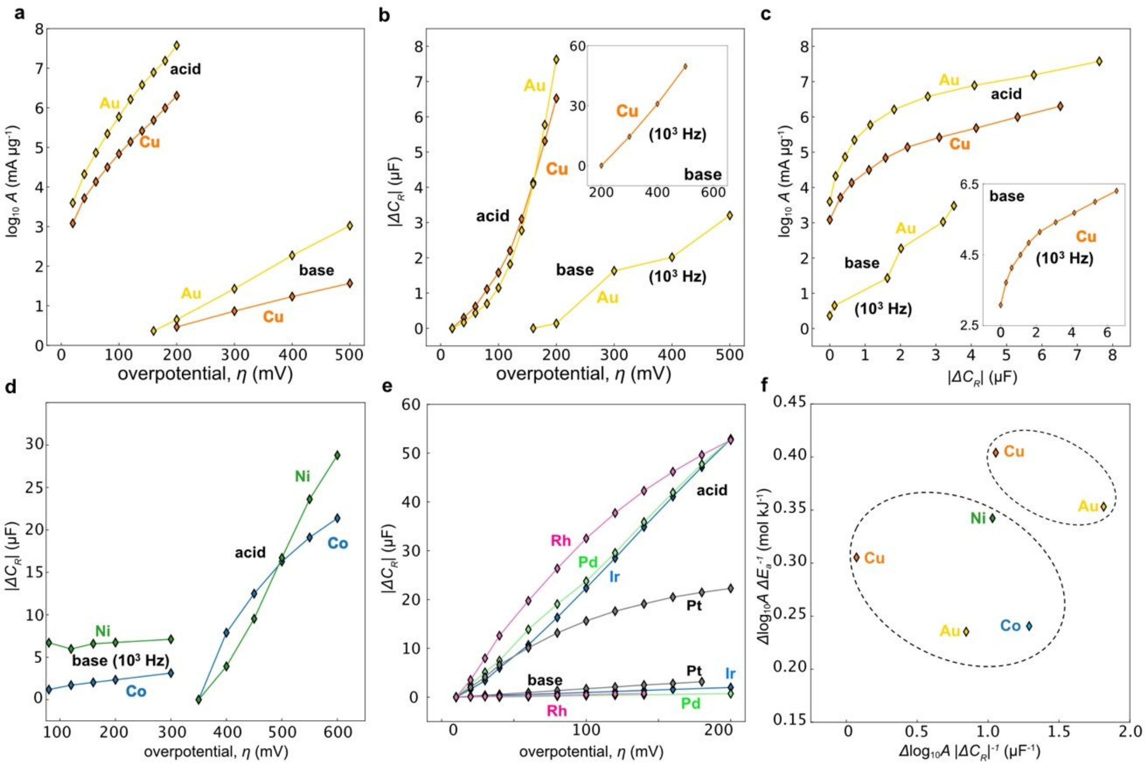

Supplementary Figure 20 |. The role of potential dependent capacitance in the hydrogen evolution kinetics. For the data in base, the GDEs were ion exchanged with hydroxide ions prior to assembly in 1M KOH. **(a-c)** Bias dependent pre-exponential factor, $\log A(\eta)$, absolute changes of the real capacitance, $|\Delta C_R(\eta)|$, and dependence of pre-exponential factor on absolute capacitance changes for Au and Cu in acid and base, respectively. **(d-e)** $|C_R(\eta)|$ for Ni and Co in acid and base and PGMs in acid and base. In acid the values are higher than for coinage metals, likely due to pseudo-capacitance related to hydrogen coverage. (**f**) $\Delta\log A(\eta)\cdot\Delta E_A(\eta)^{-1}$ vs. $\Delta\log A(\eta)\cdot|\Delta C_R(\eta)|^{-1}$ for selected catalysts. Compared to the data in Supplementary Figure 19, especially the Cu shows much higher changes in the capacitance (panel b), which might be related to dissolution or reduction of oxidized Cu.

Supplementary Table 1. Properties of commerical nanoparticle electrocatalyst dispersions.

Dispersion	Metal loading (wt %)	Carbon Support	Source	Particle Size (nm)
CuC	60	Vulcan XC-72	Fuel Cell Store	50
AgC	60	Vulcan XC-72	Fuel Cell Store	5
AuC	60	Vulcan XC-72	Fuel Cell Store	30-40
FeC	40	Vulcan XC-72	Fuel Cell Store	2
CoC	40	Ketjenblack EC-3007	Fuel Cell Store	3-4
NiC	40	Vulcan XC-72	Fuel Cell Store	40
RhC	20	Vulcan XC-72	Fuel Cell Store	2-4
IrC	40	Vulcan XC-72	Fuel Cell Store	3-5
PdC	40	Vulcan XC-72	Fuel Cell Store	5-8
PtC	70	Ketjenblack EC-300J	Fuel Cell Store	3-4

Supplementary Table 2. Working electrode catalyst loading and membranes used for each experiment.

Experiment	Membrane	GDE Metal/C + ionomer loading ($\mu g\ cm^{-2}$)	GDE Metal loading ($\mu g\ cm^{-2}$)
CuC-acid	Nafion 212	41	20
AgC-acid	Nafion 212	31	15
AuC-acid	Nafion 212	31	15
FeC-acid	Nafion 212	41	13
CoC-acid	Nafion 212	43	14
NiC-acid	Nafion 212	38	12
RhC-acid	Nafion 212	90	14
IrC-acid	Nafion 212	57	18
PdC-acid	Nafion 212	70	22
PtC-acid	Nafion 212	18	10
CuC - base	PiperION-A25	45	22
AgC - base	PiperION-A25	37	18
AuC - base	PiperION-A25	55	26
FeC - base	PiperION-A25	53	17
CoC - base	PiperION-A25	45	14
NiC - base	PiperION-A25	37	12
RhC - base	PiperION-A20	118	19
IrC - base	PiperION-A20	53	17
PdC - base	PiperION-A20	63	20
PtC - base	PiperION-A25	22	12
PtC - base - low loading	PiperION-A25	8	5
PtC - base - high loading	PiperION-A25	48	27

Supplementary Table 3. Measurement potentials and temperatures.

<table>
<tr><th>Experiment</th><th>Stabilization Potential (mV)*</th><th>Potentials measured (mV)*</th><th>Analysis temperature (ºC)**</th></tr>
<tr><td>CuC - acid</td><td>-200</td><td>-200, -180, -160, -140, -120, -100, -80, -60, -40, -20, -10</td><td rowspan="10">65, 55, 45, 35, 25</td></tr>
<tr><td>AgC - acid</td><td>-800</td><td>-800, -750, -700, -650, -600, -550, -500, -450, -400, -350, -300, -250, -200</td></tr>
<tr><td>AuC - acid</td><td>-200</td><td>-200, -180, -160, -140, -120, -100, -80, -60, -40, -20, -10</td></tr>
<tr><td>FeC - acid</td><td>-800</td><td>-800, -750, -700, -650, -600, -550, -500</td></tr>
<tr><td>CoC - acid</td><td>-800</td><td>-800, -750, -700, -650, -600, -550, -500, -450, -400, -350, -300, -250, -200, -180, -160, -140, -120, -100, -80, -60, -40, -20</td></tr>
<tr><td>NiC - acid</td><td>-800</td><td>-800, -750, -700, -650, -600, -550, -500, -450, -400, -350, -300, -250, -200, -180, -160, -140, -120, -100, -80, -60, -40, -20</td></tr>
<tr><td>RhC - acid</td><td>-200</td><td rowspan="4">-200, -180, -160, -140, -120, -100, -80, -60, -40, -20, -10</td></tr>
<tr><td>IrC - acid</td><td>-200</td></tr>
<tr><td>PdC - acid</td><td>-200</td></tr>
<tr><td>PtC - acid</td><td>-200</td></tr>
<tr><td>CuC - base</td><td rowspan="11">-600</td><td rowspan="11">-600, -500, -400, -300, -200, -160, -120, -80, -40, -20</td><td>67.5, 57.2, 45.1, 35.1, 26.7</td></tr>
<tr><td>AgC - base</td><td>55.2, 45.0, 35.1, 26.0</td></tr>
<tr><td>AuC - base</td><td>67.2, 55.4, 44.6, 35.0, 27.1</td></tr>
<tr><td>FeC - base</td><td>66.7, 56.7, 45.5, 36.2, 26.5</td></tr>
<tr><td>CoC - base</td><td>66.7, 55.1, 44.4, 35.1, 26.7</td></tr>
<tr><td>NiC - base</td><td>68.6, 55.2, 44.3, 34.3, 26.7</td></tr>
<tr><td>RhC - base</td><td>66.1, 54.6, 45.3, 36.5, 25.1</td></tr>
<tr><td>IrC - base</td><td>65.2, 53.5, 45.2, 35.2, 25.5</td></tr>
<tr><td>PdC - base</td><td>65.2, 53.8, 45.2, 35.1, 25.1</td></tr>
<tr><td>PtC - base</td><td>66.6, 55.0, 45.0, 35.0, 26.2</td></tr>
<tr><td>PtC - base - low loading</td><td>66.5, 55.2, 45.4, 34.2, 26.6</td></tr>
<tr><td>PtC - base - high loading</td><td>-200</td><td>-200, -180, -160, -140, -120, -100, -80, -60, -40, -20</td><td>66.1, 55.4, 45.7, 36.1, 26.1</td></tr>
</table>

* Experimentally, negative potential values were applied in the working electrode against the counter electrode. When discussing the data in the main manuscript the signs and currents are shown with positive values for a better representation.

** The actual temperatures used for the Arrhenius analysis of each experiment. The nominal MEA cell temperature in base was compared against another reference K-type thermocouple to account for a drift in the fuel cell test station temperature control hardware.

Supplementary References

1. Wang, S. *et al.* Electrochemical impedance spectroscopy. *Nature Reviews Methods Primers* **1**, 41 (2021).
2. Taberna, P. L., Simon, P. & Fauvarque, J. F. Electrochemical Characteristics and Impedance Spectroscopy Studies of Carbon-Carbon Supercapacitors. *Journal of The Electrochemical Society* **150**, A292 (2003).
3. Santos, E., Aradi, B., van der Heide, T. & Schmickler, W. Free energy curves for the Volmer reaction obtained from molecular dynamics simulation based on quantum chemistry. *Journal of Electroanalytical Chemistry* **954**, 118044 (2024).
4. Quaino, P. *et al.* Why DFT-Based Tight Binding Gives a Better Representation of the Potential at Metal-Solution Interfaces than DFT Does. *ChemElectroChem* **10**, e202300230 (2023).
5. Velasco-Velez, J.-J. *et al.* The structure of interfacial water on gold electrodes studied by x-ray absorption spectroscopy. *Science* **346**, 831–834 (2014).
6. Tong, Y., Lapointe, F., Thämer, M., Wolf, M. & Campen, R. K. Hydrophobic Water Probed Experimentally at the Gold Electrode/Aqueous Interface. *Angew Chem Int Ed* **56**, 4211–4214 (2017).
7. Trasatti, S. Inner later capacity in the abscence of metal-water specific interaction. *Journal of Electroanalytical Chemistry and Interfacial Electrochemistry* **91**, 293–298 (1978).
8. Agmon, N. *et al.* Protons and Hydroxide Ions in Aqueous Systems. *Chemical Reviews* **116**, 7642–7672 (2016).
9. Tuckerman, M. E., Marx, D. & Parrinello, M. The nature and transport mechanism of hydrated hydroxide ions in aqueous solution. *Nature* **417**, 925–929 (2002).
10. Cota, R., Woutersen, S. & Bakker, H. J. Accelerated Vibrational Energy Relaxation of Water in Alkaline Environments. *J. Phys. Chem. B* **125**, 11980–11986 (2021).
11. Timmer, Tielrooij, K.-J. & Bakker, H. J. Vibrational Förster transfer to hydrated protons. *The Journal of Chemical Physics* **132**, (2010).
12. Marx, D., Tuckerman, M. E., Hutter, J. & Parrinello, M. The nature of the hydrated excess proton in water. *Nature* **397**, 601–604 (1999).
13. Thämer, M., De Marco, L., Ramasesha, K., Mandal, A. & Tokmakoff, A. Ultrafast 2D IR spectroscopy of the excess proton in liquid water. *Science* **350**, 78–82 (2015).
14. He, Z., Chen, Y., Santos, E. & Schmickler, W. The Pre-exponential Factor in Electrochemistry. *Angewandte Chemie International Edition* **57**, 7948–7956 (2018).
15. Le, J.-B., Fan, Q.-Y., Li, J.-Q. & Cheng, J. Molecular origin of negative component of Helmholtz capacitance at electrified Pt(111)/water interface. *Sci. Adv.* **6**, eabb1219 (2020).
